# Arg177 and Asp159 from dog prion protein slow liquid–liquid phase separation and inhibit amyloid formation of human prion protein

**DOI:** 10.1016/j.jbc.2023.105329

**Published:** 2023-10-06

**Authors:** Xiang-Ning Li, Yuan Gao, Yang Li, Jin-Xu Yin, Chuan-Wei Yi, Han-Ye Yuan, Jun-Jie Huang, Li-Qiang Wang, Jie Chen, Yi Liang

**Affiliations:** 1 Hubei Key Laboratory of Cell Homeostasis, College of Life Sciences, TaiKang Center for Life and Medical Sciences, Wuhan University, Wuhan, China; 2 Wuhan University Shenzhen Research Institute, Shenzhen, China

**Keywords:** prion protein, protein aggregation, protein liquid–liquid phase separation, dog prion protein, prion diseases, prion disease resistance

## Abstract

Prion diseases are a group of transmissible neurodegenerative diseases primarily caused by the conformational conversion of prion protein (PrP) from α-helix-dominant cellular prion protein (PrP^C^) to β-sheet-rich pathological aggregated form of PrP^Sc^ in many mammalian species. Dogs exhibit resistance to prion diseases, but the mechanism behind the phenomenon remains poorly understood. Compared with human PrP and mouse PrP, dog PrP has two unique amino acid residues, Arg177 and Asp159. Because PrP^C^ contains a low-complexity and intrinsically disordered region in its N-terminal domain, it undergoes liquid–liquid phase separation (LLPS) *in vitro* and forms protein condensates. However, little is known about whether these two unique residues modulate the formation of PrP^C^ condensates. Here, using confocal microscopy, fluorescence recovery after photobleaching assays, thioflavin T binding assays, and transmission electron microscopy, we report that Arg177 and Asp159 from the dog PrP slow the LLPS of full-length human PrP^C^, shifting the equilibrium phase boundary to higher protein concentrations and inhibit amyloid formation of the human protein. In sharp contrast, His177 and Asn159 from the human PrP enhance the LLPS of full-length dog PrP^C^, shifting the equilibrium phase boundary to lower protein concentrations, and promote fibril formation of the canid protein. Collectively, these results demonstrate how LLPS and amyloid formation of PrP are inhibited by a single residue Arg177 or Asp159 associated with prion disease resistance, and how LLPS and fibril formation of PrP are promoted by a single residue His177 or Asn159. Therefore, Arg177/His177 and Asp159/Asn159 are key residues in modulating PrP^C^ liquid-phase condensation.

Mammalian prion protein (PrP) has two forms that are distinct in their structure and function, the cellular prion protein (PrP^C^) and its pathological aggregated form PrP^Sc^ (1, 2, 3, 4, 5, 6). Prion diseases are a group of transmissible neurodegenerative diseases primarily caused by the conformational conversion of PrP from α-helix-dominant PrP^C^ to β-sheet-rich PrP^Sc^ in many mammalian species (1, 2, 3, 4, 5, 6, 7, 8, 9, 10, 11, 12). In contrast to most mammalian species such as humans, a few mammals, including dogs, rabbits, and horses, exhibit resistance to prion diseases and are thus recognized as prion-resistant mammals (13, 14, 15, 16, 17, 18, 19, 20, 21, 22, 23, 24, 25, 26, 27, 28, 29, 30, 31, 32, 33, 34, 35, 36), but the mechanism behind the phenomenon remains unclear. Compared with PrP from most mammals including humans, PrP from dogs has two unique amino acid residues, Arg177 and Asp159, in which Asp159 suppresses the toxicity of mouse PrP in *Drosophila* (27) and has been cited as critical for encoding PrP conformational stability in prion-resistant dogs (26, 27, 28, 29, 30, 33, 34, 35). However, it is unclear whether Arg177 and Asp159 from the dog PrP regulate amyloid fibril formation of PrP.

Proteins with natively unfolded and/or low-complexity domains tend to form supramolecular assemblies called membrane-less organelles *via* liquid–liquid phase separation (LLPS) of these proteins in cells to perform key functions (37, 38, 39, 40, 41). The liquid droplets formed by biological macromolecules, called biomolecular condensates, have fusion properties (38). Because PrP^C^ contains a low-complexity and intrinsically disordered region (IDR) in its N-terminal domain (residues 23–120), it undergoes LLPS *in vitro* and forms protein condensates (42, 43, 44, 45, 46, 47, 48, 49, 50, 51). PrP^C^ liquid-phase condensation is modulated by amyloid-β oligomers, neutralizing mutations, pathological mutations, RNA (polyU RNA, crude tRNA, and yeast total RNA), and other factors (42, 43, 44, 45, 46, 47, 48, 49, 50, 51). However, it remains unknown whether Arg177 and Asp159 from the dog PrP regulate the LLPS of PrP^C^.

Here, we demonstrated that the two residues in dog PrP slowed down the LLPS of full-length human PrP^C^ and significantly inhibited amyloid formation of the human protein. In sharp contrast, His177 and Asn159 from the human PrP enhanced the LLPS of full-length dog PrP^C^ and remarkably promoted fibril formation of the canid protein. Our results provide direct evidence that LLPS and amyloid formation of PrP are inhibited by a single amino acid, Arg177 or Asp159, which is associated with prion disease resistance in dogs.

## Results

### Arg177 and Asp159 from the dog PrP slow down the LLPS of full-length human PrP^C^, but His177 and Asn159 from the human PrP enhance the LLPS of full-length dog PrP^C^

**Figure 1:**
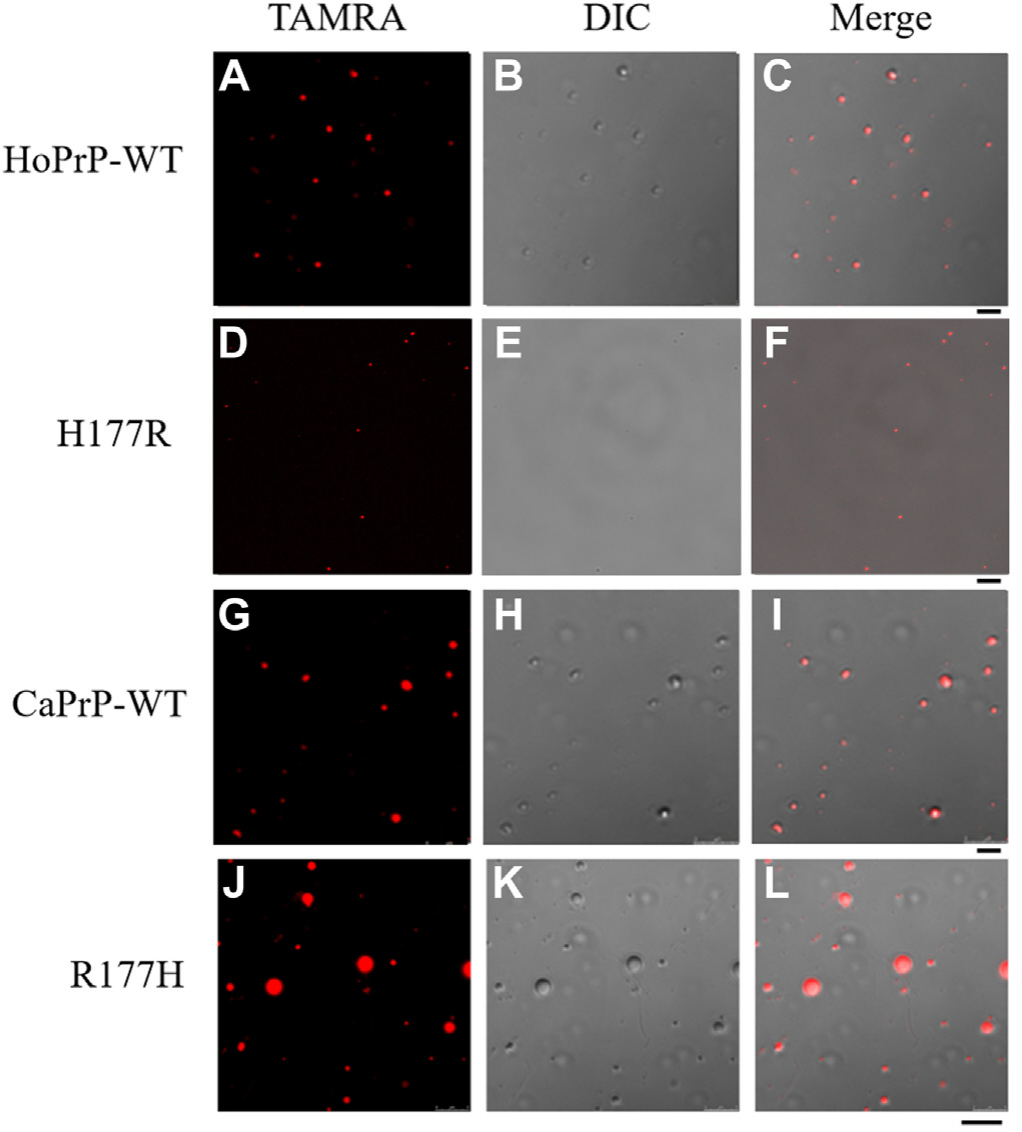
**Arg177 from the dog PrP strongly slows down liquid–liquid phase separation of full-length human PrP**^**C**^**, but His177 from the human PrP greatly enhances liquid–liquid phase separation of full-length dog PrP**^**C**^**.** Samples (50 μM) of bacterial-purified full-length wildtype human PrP^C^ (HoPrP-WT) (*A*−*C*) and its single variant H177R (*D*−*F*) as well as bacterial-purified full-length wildtype dog PrP^C^ (CaPrP-WT) (*G*−*I*) and its single variant R177H (*J*−*L*) were labeled by TAMRA (*red fluorescence*) (*A*, *D*, *G*, and *J*) and incubated with 1× PBS (pH 7.4) containing 10% (w/v) PEG 8000 on ice to induce LLPS for 10 min. Liquid droplets of the human PrP^C^ or the dog PrP^C^ were observed by differential interference contrast (DIC) confocal microscopy, with excitation at 561 nm. DIC microscopic images, *B*, *E*, *H*, and *K*. Merge, *C*, *F*, *I*, and *L*. We have replaced *D*−*F* with correct versions of H177R, in which panel *F* overlays with the DIC image in *E*. The scale bar represents 15 μm. LLPS, liquid–liquid phase separation; PrP, prion protein; PrP^C^, cellular prion protein; TAMRA, 5(6)-carboxy-tetramethylrhodamine *N*-succinimidyl ester.

**Figure 2:**
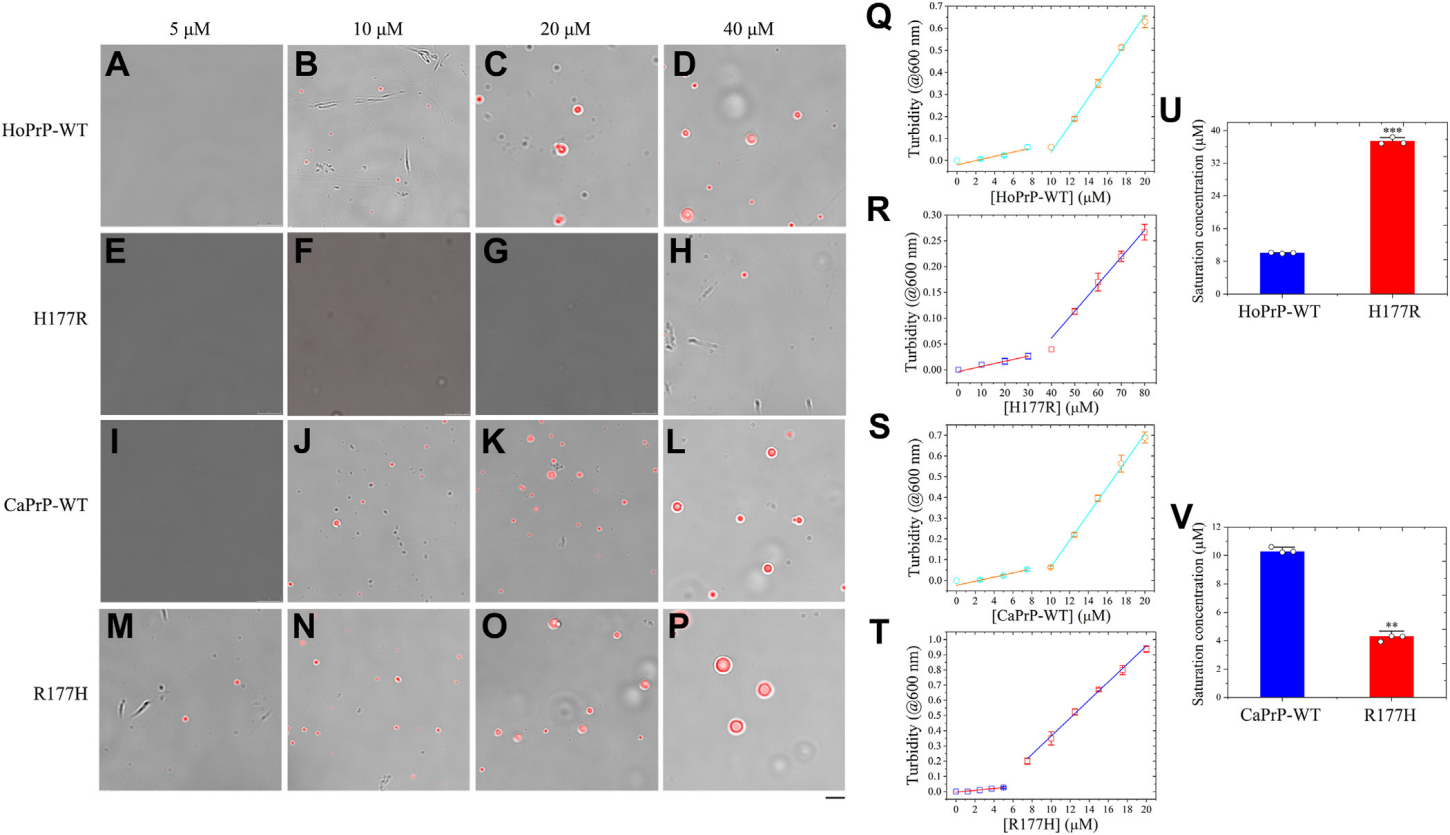
**Arg177 from the dog PrP strongly slows down the LLPS of full-length human PrP**^**C**^**, shifting the equilibrium phase boundary to a higher protein concentration.** His177 from the human PrP, however, greatly enhances the LLPS of full-length dog PrP^C^, shifting the equilibrium phase boundary to a lower protein concentration. About 5 (*A*, *E*, *I*, and *M*), 10 (*B*, *F*, *J*, and *N*), 20 (*C*, *G*, *K*, and *O*), or 40 (*D*, *H*, *L*, and *P*) μM bacterial-purified full-length wildtype human PrP^C^ (HoPrP-WT) (*A*−*D*) and its single variant H177R (*E*−*H*) as well as bacterial-purified full-length wildtype dog PrP^C^ (CaPrP-WT) (*I*−*L*) and its single variant R177H (*M*−*P*) were labeled by TAMRA (*red fluorescence*) and incubated with 1× PBS (pH 7.4) containing 10% (w/v) PEG 8000 on ice to induce LLPS for 10 min. Liquid droplets of the human PrP^C^ or the dog PrP^C^ were observed by DIC confocal microscopy, with excitation at 561 nm. *A*−*P*, the merged images of the TAMRA images in Fig. S2, *A*−*P* and the DIC microscopic images in Fig. S3, *A*−*P*. The scale bar represents 10 μm. *Q*−*T*, the turbidity of PrP^C^ condensates was measured at 600 nm and 25 °C. The dependence of turbidity changes for LLPS of HoPrP-WT (*Q*), its single variant H177R (*R*), CaPrP-WT (*S*), or its single variant R177H (*T*) on the concentration of HoPrP-WT ([HoPrP-WT]), H177R ([H177R]), CaPrP-WT ([CaPrP-WT]), or R177H ([R177H]) was expressed as mean ± SD (with error bars) of values obtained in three independent experiments. Representative calculation based on turbidity measurements to determine saturation concentration of the wildtype protein (HoPrP-WT or CaPrP-WT) (*open circle*) or the single variant (H177R or R177H) (*open square*). The *orange* and *red lines* are drawn through data points indicating the absence of LLPS, whereas the *cyan* and *blue lines* are drawn through data points in which robust LLPS of the wildtype protein (HoPrP-WT or CaPrP-WT) and the single variant (H177R or R177H) occurs, respectively. The concentration of protein at which these two lines intersect is an estimation of the saturation concentration. *U* and *V*, saturation concentrations of the wildtype proteins (HoPrP-WT and CaPrP-WT, *blue*) and the single variants (H177R and R177H, *red*) (*open black circles* shown in scatter plots) were expressed as the mean ± SD (with error bars) of values obtained in three independent experiments. *U*, H177R, *p* = 0.00047; *V*, R177H, *p* = 0.0043. Statistical analyses were performed using the Student's *t* test. Values of *p* < 0.05 indicate statistically significant differences. The following notation is used throughout: ∗*p* < 0.05; ∗∗*p* < 0.01; and ∗∗∗*p* < 0.001 relative to control (the saturation concentration for wildtype PrP^C^). DIC, differential interference contrast; LLPS, liquid–liquid phase separation; PrP, prion protein; PrP^C^, cellular prion protein; TAMRA, 5(6)-carboxy-tetramethylrhodamine *N*-succinimidyl ester.

Human PrP and mouse PrP have an Asn at position 159 and a His at position 177, but dog PrP has an Asp or Glu at position 159 and an Arg at position 177 (Fig. S1). Given that the presence of Arg177 and Asp159 in dogs causes unique charge distribution patterns on the front and back sides of dog PrP^C^, which might in turn protect dogs from getting prion diseases (27, 28, 29, 52, 53), we predicted that the two residues in dog PrP might regulate the LLPS of human PrP^C^ by increasing the saturation concentration for PrP condensation. We next used confocal microscopy and fluorescence recovery after photobleaching (FRAP) (43, 44, 46, 47, 49, 50, 51) to test this hypothesis. Bacterial-purified full-length wildtype human PrP^C^, its single variant H177R, full-length wildtype dog PrP^C^, and its single variant R177H, labeled by 5(6)-carboxy-tetramethylrhodamine *N*-succinimidyl ester (TAMRA, red fluorescence) and incubated with 1× PBS (pH 7.4) containing 10% (w/v) PEG 8000 on ice, underwent LLPS *in vitro* and formed protein condensates (Fig. 1, *A*−*L*). Here, PEG 8000 was used to mimic cellular crowded environments accurately. Wildtype human PrP^C^ formed abundant liquid droplets, and protein condensates formed by H177R PrP^C^ became much less than those formed by the wildtype (Fig. 1, *A*−*F*). Notably, Arg177 from the dog PrP strongly slowed down *in vitro* LLPS of full-length human PrP^C^ (Fig. 1, *A*−*F*). Wildtype dog PrP^C^ also formed abundant liquid droplets, and protein condensates formed by R177H PrP^C^ became much larger than those formed by the wildtype (Fig. 1, *G*−*L*). Notably, His177 from the human PrP greatly enhanced *in vitro* LLPS of full-length dog PrP^C^ (Fig. 1, *G*−*L*). In total, 5 μM wildtype human PrP^C^ did not form any liquid droplets, the wildtype at 10 μM did produce a few liquid droplets, and 20 or 40 μM wildtype human PrP^C^ formed abundant liquid droplets in PBS buffer containing 10% PEG 8000 on ice (Figs. 2, S2, and S3, *A*−*D*). However, 10 or 20 μM H177R PrP^C^ did not form any liquid droplets, and 40 μM H177R PrP^C^ did produce a few liquid droplets in the same buffer on ice (Figs. 2, S2, and S3, *E*−*H*). In total, 5 μM wildtype dog PrP^C^ did not form any liquid droplets, the wildtype at 10 μM did produce a few liquid droplets, and 20 or 40 μM wildtype dog PrP^C^ formed abundant liquid droplets in PBS buffer containing 10% PEG 8000 on ice (Figs. 2, S2, and S3, *I*−*L*). However, 5 μM R177H PrP^C^ did form a few liquid droplets, 10, 20, or 40 μM R177H PrP^C^ formed abundant liquid droplets, and liquid droplets gradually increased their size with the increase of concentration of R177H (Figs. 2, S2, and S3, *M*−*P*). The TAMRA images (Fig. S2, *A*−*P*) and the differential interference contrast microscopic images (Fig. S3, *A*−*P*) are of the same field of view and are mostly overlapping proved by the merged images (Fig. 2, *A*−*P*) for the same experiments collected at the same focal plane. A generally accepted measure of protein propensity for LLPS is the saturation concentration (54, 55, 56, 57, 58). To assess the effects of Arg177 from the dog PrP and His177 from the human PrP on PrP^C^ propensity for LLPS, we determined saturation concentrations of wildtype human/dog PrP^C^ and its single variant H177R/R177H by measuring the turbidity of PrP^C^ condensates at 600 nm as a function of the concentration of PrP^C^ (Fig. 2, *Q*−*T*). The turbidity of a solution does not increase when no LLPS occurs, based on the absorbance at 600 nm (Fig. 2, *Q*−*T*). We then compared the saturation concentrations of the wildtype proteins and the single variants (Fig. 2, *U* and *V*). These data clearly demonstrate that the saturation concentration, the concentration above which PrP^C^ starts to form liquid droplets, was 10.00 ± 0.13 μM for wildtype human PrP^C^, 37.36 ± 0.90 μM for its single variant H177R, 10.28 ± 0.31 μM for wildtype dog PrP^C^, and 4.32 ± 0.37 μM for its single variant R177H (Fig. 2, *U* and *V*). Unexpectedly, the saturation concentration of wildtype dog PrP^C^ is equal to the wildtype human protein (*p* = 0.39), although we expected dog PrP^C^ to be higher than human PrP^C^ as the canid protein has both Arg177 and Asp159. We comment on why the values for these two proteins are the same as follows. Compared with PrP from humans and mice, PrP from dogs has eight unique amino acid residues in its N-terminal IDR, such as Gly100 and Asn103 (Fig. S1), which might also regulate the LLPS of dog PrP^C^. Importantly, we found that the saturation concentration of H177R (*p* = 0.00047) was significantly higher than that of wildtype human PrP^C^ (Fig. 2*U*), but the saturation concentration of R177H (*p* = 0.0043) was significantly lower than that of wildtype dog PrP^C^ (Fig. 2*V*). Together, the data showed that Arg177 from the dog PrP strongly slows down the LLPS of full-length human PrP^C^, shifting the equilibrium phase boundary to a higher protein concentration. In sharp contrast, His177 from the human PrP greatly enhances the LLPS of full-length dog PrP^C^, shifting the equilibrium phase boundary to a lower protein concentration.

**Figure 3:**
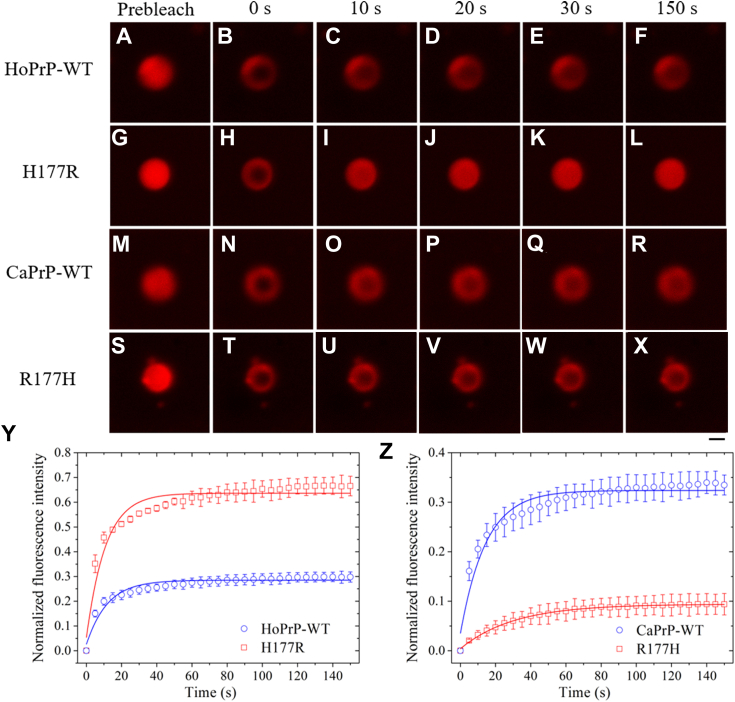
**Arg177 from the dog PrP enhances fluorescence recovery and negatively modulates the LLPS of full-length human PrP**^**C**^**.** His177 from the human PrP, however, decreases fluorescence recovery and positively modulates the LLPS of full-length dog PrP^C^. *A*−*X*, FRAP analysis on the selected liquid droplets of 50 μM bacterial-purified full-length wildtype human PrP^C^ (HoPrP-WT) (*A*−*F*) and its single variant H177R (*G*−*L*) as well as bacterial-purified full-length wildtype dog PrP^C^ (CaPrP-WT) (*M*−*R*) and its single variant R177H (*S*−*X*) labeled by TAMRA (*red fluorescence*) before (prebleach, *A*, *G*, *M*, and *S*), during (0 s, *B*, *H*, *N*, and *T*), and after photobleaching (10 s, *C*, *I*, *O*, and *U*; 20 s, *D*, *J*, *P*, and *V*; 30 s, *E*, *K*, *Q*, and *W*; 150 s, *F*, *L*, *R*, and *X*). The internal photobleaching is marked by a *black square*. Full-length PrP was incubated with 1× PBS (pH 7.4) containing 10% (w/v) PEG 8000 on ice to induce LLPS for 10 min, and liquid droplets were observed by confocal microscopy, with excitation at 561 nm. The scale bars represent 1 μm. The images in *P*, *Q*, and *R* almost did not change over time, and the images in *U*, *V*, *W*, and *X* almost did not change over time. *Y* and *Z*, normalized kinetics of fluorescence recovery data of the wildtype protein (HoPrP-WT or CaPrP-WT) (*open blue circle*) and the single variant (H177R or R177H) (*open red square*) obtained from FRAP intensity. The normalized fluorescence intensity is expressed as the mean ± SD of the values obtained in three independent experiments. The *solid blue* or *red lines* show the best single exponential fit for the fluorescence intensity–time curves. FRAP of phase-separated droplets of HoPrP-WT, its single variant H177R (*Y*), CaPrP-WT, or its single variant R177H (*Z*) revealed a fluorescence recovery rate of (8.42 ± 0.89) × 10^−2^ s^−1^, (9.27 ± 1.01) × 10^−2^ s^−1^, (6.94 ± 0.68) × 10^−2^ s^−1^, or (3.28 ± 0.09) × 10^−2^ s^−1^ with a (25.9 ± 1.4)%, (58.2 ± 3.2)%, (28.8 ± 1.4)%, or (9.00 ± 0.11)% fluorescence recovery within 150 s. All FRAP experiments were repeated three times, and the results were reproducible. FRAP, fluorescence recovery after photobleaching; LLPS, liquid–liquid phase separation; PrP, prion protein; PrP^C^, cellular prion protein; TAMRA, 5(6)-carboxy-tetramethylrhodamine *N*-succinimidyl ester.

We then investigated and evaluated the dynamics of *in vitro* phase-separated droplets of wildtype human/dog PrP^C^ and its single variant H177R/R177H by FRAP (Fig. 3, *A*−*Z*). FRAP of phase-separated droplets of wildtype human PrP^C^ revealed fluorescence recovery of (25.9 ± 1.4)% with a fluorescence recovery rate of (8.42 ± 0.89) × 10^−2^ s^−1^ within 150 s (Fig. 3*Y*). FRAP of phase-separated droplets of H177R PrP^C^, however, revealed much higher fluorescence recovery, (58.2 ± 3.2)%, with a similar fluorescence recovery rate of (9.27 ± 1.01) × 10^−2^ s^−1^ within 150 s (Fig. 3*Y*). FRAP of phase-separated droplets of wildtype dog PrP^C^ revealed fluorescence recovery of (28.8 ± 1.4)% with a fluorescence recovery rate of (6.94 ± 0.68) × 10^−2^ s^−1^ within 150 s (Fig. 3*Z*). FRAP of phase-separated droplets of R177H PrP^C^, however, revealed much lower fluorescence recovery, (9.00 ± 0.11)%, with a lower fluorescence recovery rate of (3.28 ± 0.09) × 10^−2^ s^−1^ within 150 s (Fig. 3*Z*). The aforementioned experiments help drive the narrative that Arg177 from the dog PrP enhances fluorescence recovery and negatively modulates the LLPS of full-length human PrP^C^. His177 from the human PrP, however, decreases fluorescence recovery and positively modulates the LLPS of full-length dog PrP^C^.

**Figure 4:**
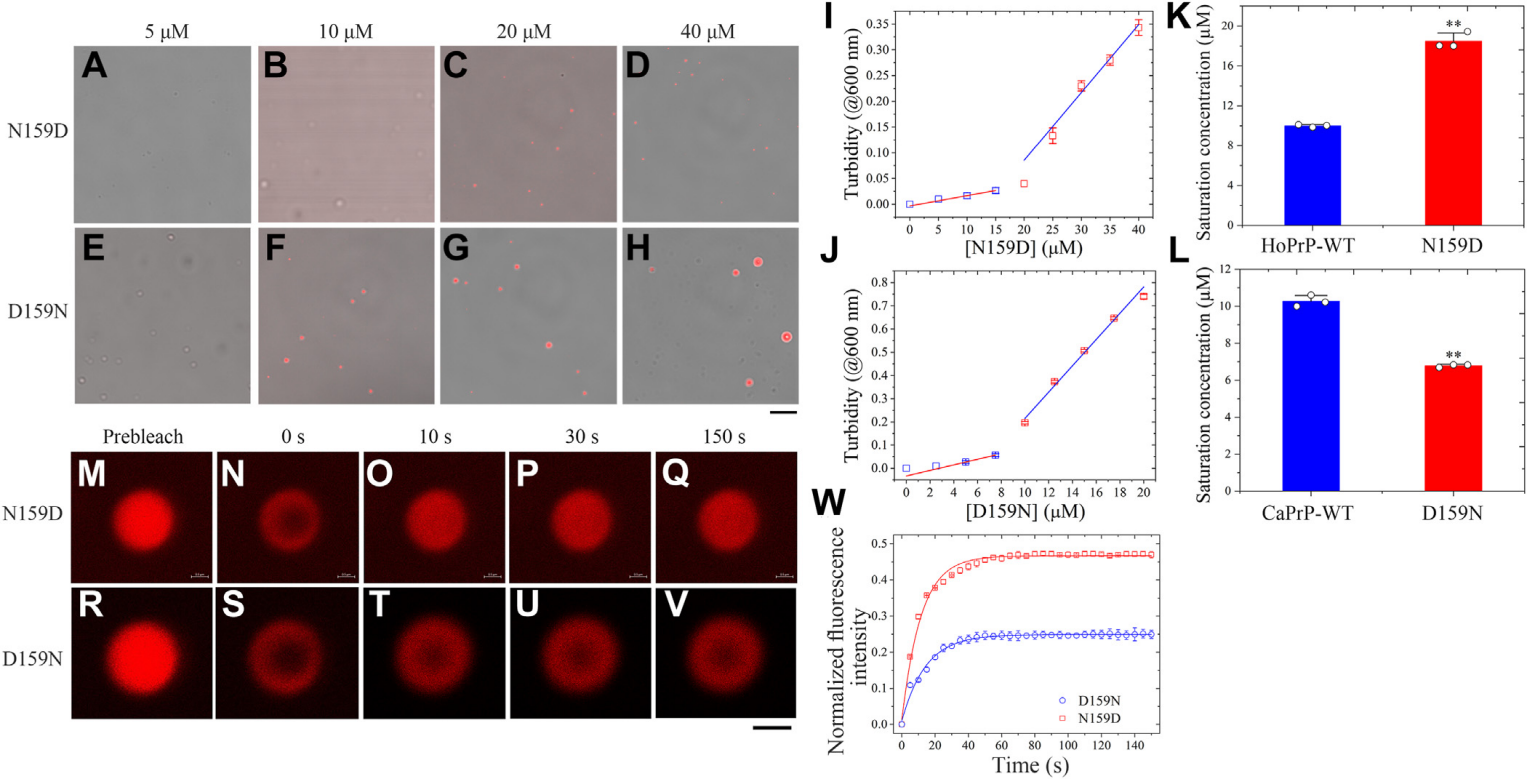
**Asp159 from the dog PrP slows down the LLPS of full-length human PrP**^**C**^**, shifting the equilibrium phase boundary to a higher protein concentration.** Asn159 from the human PrP, however, enhances the LLPS of full-length dog PrP^C^, shifting the equilibrium phase boundary to a lower protein concentration. About 5 (*A* and *E*), 10 (*B* and *F*), 20 (*C* and *G*), or 40 (*D* and *H*) μM bacterial-purified single variant N159D of full-length human PrP^C^ (*A*−*D*) and single variant D159N of full-length dog PrP^C^ (*E*−*H*) were labeled by TAMRA (*red fluorescence*) and incubated with 1× PBS (pH 7.4) containing 10% (w/v) PEG 8000 on ice to induce LLPS for 10 min. Liquid droplets of the human PrP^C^ or the dog PrP^C^ were observed by DIC confocal microscopy, with excitation at 561 nm. We have replaced *H* with a correct version of D159N. The scale bar represents 10 μm. *I* and *J*, the turbidity of PrP^C^ condensates was measured at 600 nm and 25 °C. The dependence of turbidity changes for LLPS of the single variant N159D of HoPrP-WT (*I*) or the single variant D159N of CaPrP-WT (*J*) on the concentration of N159D ([N159D]) or D159N ([D159N]) was expressed as mean ± SD (with error bars) of values obtained in three independent experiments. Representative calculation based on turbidity measurements to determine saturation concentration of the single variant (N159D or D159N) (*open square*). The *red line* is drawn through data points indicating the absence of LLPS, whereas the *blue line* is drawn through data points in which robust LLPS of the single variant (N159D or D159N) occurs. The concentration of protein at which these two lines intersect is an estimation of the saturation concentration. *K* and *L*, saturation concentrations of the wildtype proteins (HoPrP-WT and CaPrP-WT, *blue*) and the single variants (N159D and D159N, *red*) (*open black circles* shown in scatter plots) were expressed as the mean ± SD (with error bars) of values obtained in three independent experiments. *K*, N159D, *p* = 0.0030; *L*, D159N, *p* = 0.0017. Statistical analyses were performed using the Student's *t* test. Values of *p* < 0.05 indicate statistically significant differences. The following notation is used throughout: ∗*p* < 0.05; ∗∗*p* < 0.01; and ∗∗∗*p* < 0.001 relative to control (the saturation concentration for wildtype PrP^C^). *M*−*V*, FRAP analysis on the selected liquid droplets of 50 μM bacterial-purified single variant N159D of human PrP^C^ (*M*−*Q*) and single variant D159N of dog PrP^C^ (*R*−*V*) labeled by TAMRA (*red fluorescence*) before (prebleach, *M* and *R*), during (0 s, *N* and *S*), and after photobleaching (10 s, *O* and *T*; 30 s, *P* and *U*; 150 s, *Q* and *V*). The internal photobleaching is marked by a *black square*. The scale bars represent 1 μm. The images in *O*, *P*, and *Q* almost did not change over time, and the images in *T*, *U*, and *V* almost did not change over time. *W*, normalized kinetics of fluorescence recovery data of D159N (*open blue circle*) and N159D (*open red square*) obtained from FRAP intensity. The normalized fluorescence intensity is expressed as the mean ± SD of the values obtained in three independent experiments. The *solid blue lines* or *red lines* show the best single exponential fit for the fluorescence intensity–time curves. FRAP of phase-separated droplets of single variant N159D of human PrP^C^ or single variant D159N of dog PrP^C^ revealed a fluorescence recovery rate of (8.70 ± 0.36) × 10^−2^ s^−1^ or (7.03 ± 0.34) × 10^−2^ s^−1^ with a (45.2 ± 1.0)% or (23.8 ± 0.6)% fluorescence recovery within 150 s. All FRAP experiments were repeated three times, and the results were reproducible. DIC, differential inference contrast; FRAP, fluorescence recovery after photobleaching; LLPS, liquid–liquid phase separation; PrP, prior protein; PrP^C^, cellular prion protein; TAMRA, 5(6)-carboxy-tetramethylrhodamine *N*-succinimidyl ester.

Because dogs are resistant to prion infection, partly because of the presence of Asp159 in their PrP (26, 27, 28, 29, 30, 33, 34, 35), and Asp159 suppresses the toxicity of mouse PrP in *Drosophila* (27), we chose to investigate the regulation mechanism of Asp159 on PrP^C^ liquid-phase condensation. We used two mutations at codon 159, bacterial-purified N159D of human PrP^C^ and D159N of dog PrP^C^, labeled by TAMRA (red fluorescence) and incubated with 1× PBS (pH 7.4) containing 10% (w/v) PEG 8000 on ice. We did observe the LLPS of N159D and D159N PrP^C^ by confocal microscopy (Fig. 4, *A*−*H*). In total, 10 μM N159D PrP^C^ did not form any liquid droplets, and 20 or 40 μM N159D PrP^C^ formed abundant liquid droplets in PBS buffer containing 10% PEG 8000 on ice (Fig. 4, *A*−*D*). In total, 5 μM D159N PrP^C^ did not form any liquid droplets, and 10, 20, or 40 μM D159N PrP^C^ formed abundant liquid droplets (Fig. 4, *E*−*H*). To assess the effects of Asp159 from the dog PrP and Asn159 from the human PrP on PrP^C^ propensity for LLPS, we determined saturation concentrations of N159D and D159N PrP^C^ by measuring the turbidity of PrP^C^ condensates at 600 nm as a function of the concentration of PrP^C^ (Fig. 4, *I* and *J*). These data clearly demonstrate that the saturation concentration was 18.49 ± 0.82 μM for N159D of human PrP^C^ and 6.79 ± 0.08 μM for D159N of dog PrP^C^ (Fig. 4, *K* and *L*). Importantly, we found that the saturation concentration of N159D (*p* = 0.0030) was significantly higher than that of wildtype human PrP^C^ (Fig. 4*K*), but the saturation concentration of D159N (*p* = 0.0017) was significantly lower than that of wildtype dog PrP^C^ (Fig. 4*L*). Together, the data showed that Asp159 from the dog PrP slows down the LLPS of full-length human PrP^C^, shifting the equilibrium phase boundary to a higher protein concentration. In sharp contrast, Asn159 from the human PrP promotes the LLPS of full-length dog PrP^C^, shifting the equilibrium phase boundary to a lower protein concentration.

We then investigated and evaluated the dynamics of *in vitro* phase-separated droplets of the two single variants, N159D and D159N PrP^C^, by FRAP (Fig. 4, *M*−*W*). Compared with wildtype human PrP^C^ (Fig. 3*Y*), FRAP of phase-separated droplets of N159D PrP^C^ revealed much higher fluorescence recovery, (45.2 ± 1.0)%, with a similar fluorescence recovery rate of (8.70 ± 0.36) × 10^−2^ s^−1^ within 150 s (Fig. 4*W*). Compared with wildtype dog PrP^C^ (Fig. 3*Z*), however, FRAP of phase-separated droplets of D159N PrP^C^ revealed remarkably lower fluorescence recovery, (23.8 ± 0.6)%, with a similar fluorescence recovery rate of (7.03 ± 0.34) × 10^−2^ s^−1^ within 150 s (Fig. 4*W*). The aforementioned experiments help drive the narrative that Asp159 from the dog PrP enhances fluorescence recovery and negatively modulates the LLPS of full-length human PrP^C^. Asn159 from the human PrP, however, decreases fluorescence recovery and positively modulates the LLPS of full-length dog PrP^C^.

Altogether, these data strongly suggest that Arg177/Asp159 from the dog PrP and His177/Asn159 from the human PrP control liquidity. H177R and N159D enhance but R177H and D159N reduce PrP^C^ mobility. Importantly, Arg177 and Asp159 from the dog PrP slow down the LLPS of full-length human PrP^C^ *via* increasing the saturation concentration for PrP condensation, but His177 and Asn159 from the human PrP promote the LLPS of full-length dog PrP^C^ *via* decreasing the saturation concentration for PrP condensation. Therefore, Arg177/Asp159 from the dog PrP and His177/Asn159 from the human PrP are key amino acid residues in modulating PrP^C^ liquid-phase condensation.

### Arg177 and Asp159 from the dog PrP inhibit amyloid formation of the human protein, but His177 and Asn159 from the human PrP promote fibril formation of the canid protein

**Figure 5:**
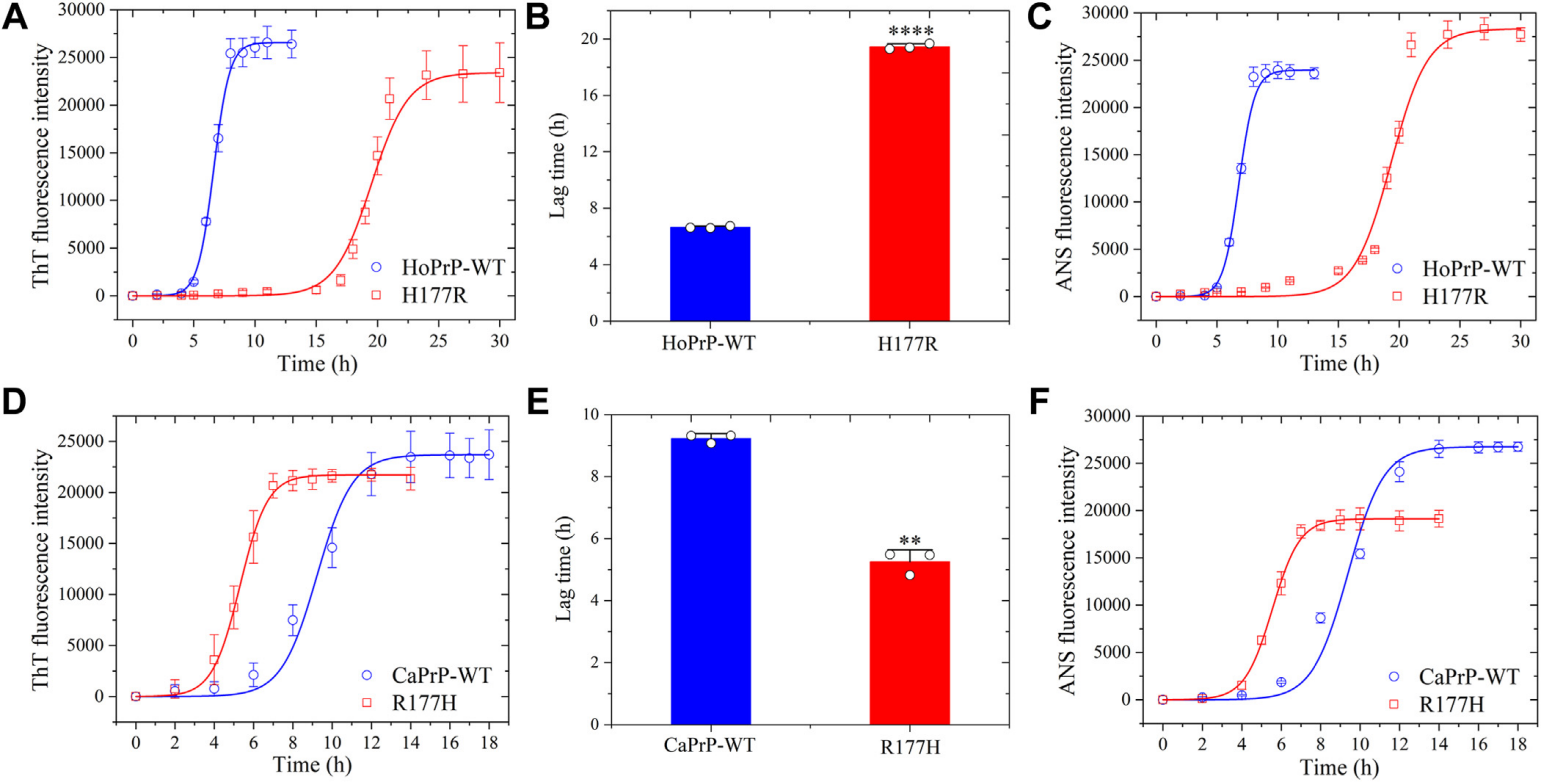
**Arg177 from the dog PrP significantly inhibits amyloid formation of human PrP, but His177 from the human PrP greatly enhances fibril formation of dog PrP.** Samples (20 μM) of HoPrP-WT (*blue*) and its single variant H177R (*red*) (*A*−*C*) as well as CaPrP-WT (*blue*) and its single variant R177H (*red*) (*D*−*F*) were incubated in 1× PBS (pH 7.4) containing 2 M GdnHCl with agitation at 220 rpm and then analyzed by thioflavin T (ThT) binding assay (*A*, *B*, *D*, and *E*) and 8-anilino-1-naphthalene-sulfonic acid (ANS) binding assay (*C* and *F*). *A*, *C*, *D*, and *F*, the ThT and ANS fluorescence intensities were expressed as the mean ± SD (with error bars) of values obtained in three independent experiments. The *solid lines* show the best exponential fit for the ThT/ANS intensity–time curves. *B* and *E*, the fibril formation lag time in the wildtype proteins (HoPrP-WT and CaPrP-WT, *blue*) and the single variants (H177R and R177H, *red*) (*open black circles* shown in scatter plots) was determined by fitting ThT fluorescence intensity *versus* time to a sigmoidal equation and was expressed as the mean ± SD (with error bars) of values obtained in three independent experiments. *B*, H177R, *p* = 0.000034; *E*, R177H, *p* = 0.0012. Statistical analyses were performed using the Student's *t* test. Values of *p* < 0.05 indicate statistically significant differences. The following notation is used throughout: ∗*p* < 0.05; ∗∗*p* < 0.01; ∗∗∗*p* < 0.001; and ∗∗∗∗*p* < 0.0001 relative to control (the lag time for wildtype PrP) (*B* and *E*). ANS, 8-anilino-1-naphthalene-sulfonic acid; GdnHCl, guanidine hydrochloride; PrP, prion protein; ThT, thioflavin T.

**Figure 6:**
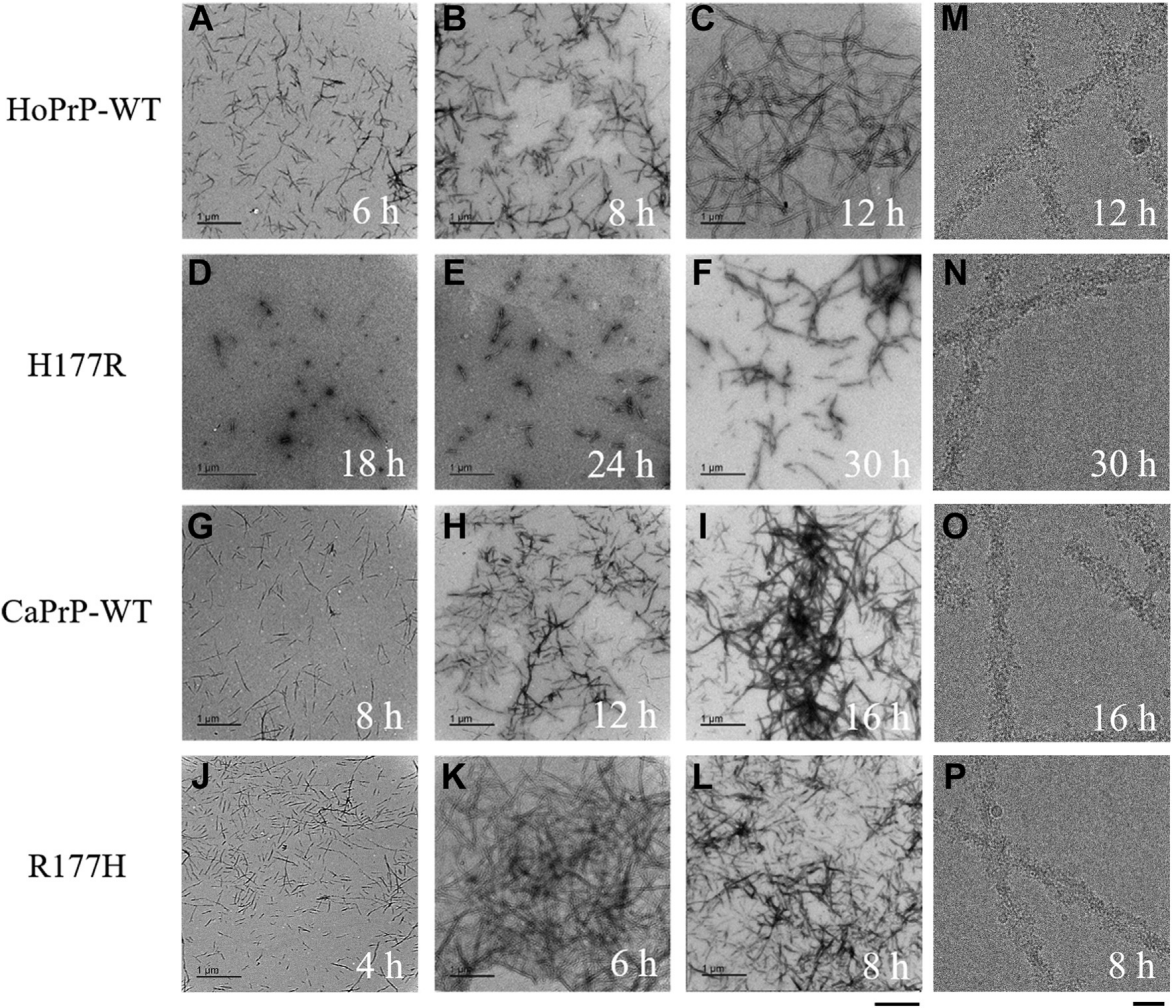
**Time-course negative-staining TEM images show that Arg177 from the dog PrP strongly inhibits amyloid formation of human PrP, but His177 from the human PrP greatly enhances fibril formation of dog PrP.** Samples (20 μM) of HoPrP-WT (*A*−*C* and *M*) and its single variant H177R (*D*−*F* and *N*) as well as CaPrP-WT (*G*−*I* and *O*) and its single variant R177H (*J*−*L* and *P*) were incubated in 1× PBS (pH 7.4) containing 2 M GdnHCl incubated for 4 (*J*), 6 (*A* and *K*), 8 (*B*, *G*, *L*, and *P*), 12 (*C*, *H*, and *M*), 16 (*I* and *O*), 18 (*D*), 24 (*E*), and 30 (*F* and *N*) with agitation at 220 rpm. *A*−*L*, a 2% (w/v) uranyl acetate solution was used for staining the fibrils negatively. Scale bar represents 500 nm. *M*−*P*, cryo-EM micrographs of amyloid fibrils of HoPrP-WT (*M*), H177R (*N*), CaPrP-WT (*O*), and R177H (*P*). Scale bar represents 40 nm. GdnHCl, guanidine hydrochloride; PrP, prionprotein; TEM, transmission electron microscopy.

Given that Arg177 and Asp159 from the dog PrP regulate *in vitro* LLPS of human PrP^C^ (Figure 1, Figure 2, Figure 3, Figure 4), we predicted that these two unique amino acid residues might also regulate the aggregation of human PrP by increasing the lag time of its fibrillization. We next used thioflavin T (ThT) binding assay, 8-anilino-1-naphthalene-sulfonic acid (ANS) binding assay, SDS-PAGE of sarkosyl-soluble PrP, and transmission electron microscopy (TEM) (7, 8, 19, 44) to test this hypothesis. We produced amyloid fibrils from bacterial-purified full-length wildtype human PrP, its single variant H177R, full-length wildtype dog PrP^C^, and its single variant R177H, by incubating the purified proteins (20 μM) in 1× PBS (pH 7.4) containing 2 M guanidine hydrochloride (GdnHCl) with agitation at 220 rpm for 13, 30, 18, and 14 h, respectively, and the kinetics of fibril formation was then analyzed by ThT binding assay and ANS binding assay (Fig. 5, *A*−*F*) combined with SDS-PAGE of sarkosyl-soluble PrP (Fig. S4, *A*−*C*). We have shown error bars for the ThT/ANS assay data points as well as individual data points overlayed on the bar graphs (Fig. 5, *A*−*F*) and have provided an indication of the reproducibility of the results in Fig. S4, *A*−*C*. Notably, Arg177 from the dog PrP significantly inhibited amyloid formation of the human protein (Figs. 5, *A*−*C*, and S4, *A*−*C*). The lag time of H177R fibrillization (Fig. 5*B*: 19.46 ± 0.20 h, *p* = 0.000034; Fig. S4, *A*−*C*: over 20 h) was significantly higher than that of wildtype human PrP (Fig. 5*B*: 6.66 ± 0.08 h; Fig. S4, *A*−*C*: over 7 h), indicating that the H177R mutation delays the kinetics of fibril formation of human PrP. In sharp contrast, His177 from the human PrP greatly enhanced fibril formation of the canid protein (Figs. 5, *D*−*F*, and S4, *A*−*C*). Compared with the fibril formation lag time in wildtype dog PrP (Fig. 5*E*: 9.24 ± 0.15 h; Fig. S4, *A*−*C*: over 10 h), the lag time of fibril formation was significantly decreased in R177H (Fig. 5*E*: 5.26 ± 0.38 h, *p* = 0.0012; Fig. S4, *A*−*C*: over 6 h), indicating that the R177H mutation promotes the kinetics of fibril formation of dog PrP. Importantly, time-course negative-staining TEM images and cryo-EM images showed that wildtype human PrP, its single variant H177R, wildtype dog PrP, and its single variant R177H all formed homogeneous amyloid fibrils (Fig. 6, *A*−*P*) and that Arg177 from the dog PrP strongly inhibited amyloid formation of the human protein, but His177 from the human PrP greatly enhanced fibril formation of the canid protein (Fig. 6, *A*−*L*). In total, 20 μM wildtype human PrP did produce short amyloid fibrils in PBS buffer containing 2 M GdnHCl incubated for 6 and 8 h (Fig. 6, *A* and *B*) and formed abundant long and branched amyloid fibrils when incubated for 12 h (Fig. 6*C*). However, 20 μM H177R PrP did not form any amyloid fibrils in the same buffer incubated for 18 h (Fig. 6*D*) and did produce a few amyloid fibrils when incubated for a much longer time (24 h) (Fig. 6*E*) and formed abundant amyloid fibrils when incubated for 30 h (Fig. 6*F*). In total, 20 μM wildtype dog PrP did produce short amyloid fibrils in PBS buffer containing 2 M GdnHCl incubated for 8 h (Fig. 6*G*) and formed abundant amyloid fibrils when incubated for 12 and 16 h (Fig. 6, *H* and *I*). However, 20 μM R177H PrP did form short amyloid fibrils in the same buffer incubated for a remarkably shorter time (4 h) (Fig. 6*J*) and formed abundant branched amyloid fibrils when incubated for 6 and 8 h (Fig. 6, *K* and *L*).

We next chose to investigate the regulation mechanism of Asp159 on PrP aggregation. We produced amyloid fibrils from bacterial-purified N159D of human PrP and D159N of dog PrP, by incubating the purified proteins (20 μM) in PBS buffer containing 2 M GdnHCl with agitation at 220 rpm for 24 and 14 h, respectively, and the kinetics of fibril formation was then analyzed by ThT binding assay and SDS-PAGE of sarkosyl-soluble PrP (Figs. S4, *A*−*C* and S5*A*). Notably, Asp159 from the dog PrP significantly inhibited amyloid formation of the human protein (Figs. S4, *A*−*C* and S5*A*). The lag time of N159D fibrillization (Fig. S5*A*: 14.70 ± 0.07 h, *p* = 0.00011; Fig. S4, *A*−*C*: over 14 h) was significantly higher than that of wildtype human PrP (Fig. 5*B*: 6.66 ± 0.08 h; Fig. S4, *A*−*C*: over 7 h), indicating that the N159D mutation also delays the kinetics of fibril formation of human PrP. In sharp contrast, Asn159 from the human PrP greatly enhanced fibril formation of the canid protein (Figs. S4, *A*−*C* and S5*A*). Compared with the fibril formation lag time in wildtype dog PrP (Fig. 5*E*: 9.24 ± 0.15 h; Fig. S4, *A*−*C*: over 10 h), the lag time of fibril formation was significantly decreased in D159N (Fig. S5*A*: 6.73 ± 0.08 h, *p* = 0.00039; Fig. S4, *A*−*C*: over 6 h), indicating that the D159N mutation also promotes the kinetics of fibril formation of dog PrP. Importantly, time-course negative-staining TEM images and cryo-EM images showed that N159D of human PrP and D159N of dog PrP both formed homogeneous amyloid fibrils (Fig. S5, *B*−*I*) and that Asp159 from the dog PrP strongly inhibited amyloid formation of the human protein, but Asn159 from the human PrP greatly enhanced fibril formation of the canid protein (Fig. S5, *B*−*G*). In total, 20 μM N159D PrP did not form any amyloid fibrils in PBS buffer containing 2 M GdnHCl incubated for 10 h (Fig. S5*C*) and did produce abundant amyloid fibrils when incubated for a remarkably longer time (18 h) (Fig. S5*D*). In total, 20 μM D159N PrP did form short amyloid fibrils in the same buffer incubated for a shorter time (6 h) (Fig. S5*F*) and formed abundant amyloid fibrils when incubated for 10 h (Fig. S5*G*).

Moreover, we wanted to determine if the primary effects of the mutations are on the nucleation phase or the elongation phase of fibril formation of PrP. The observed effects of the mutations on saturation concentration of PrP^C^ suggest effects on nucleation step (Figs. 2 and 4). We then compared the observed effects on fibrillization reactions that were seeded with preformed PrP fibrils, where the nucleation phase was largely bypassed (Fig. S6, *A*−*F*). Here, the preformed seeds match the sequence of the monomeric substrate in each case in order to avoid effects of mismatches. Compared with the fibril formation lag time in H177R, R177H, N159D, and D159N in the absence of preformed seed fibrils (Fig. 5*B*: 19.46 ± 0.20 h; Fig. 5*E*: 5.26 ± 0.38 h; Fig. S5*A*: 14.70 ± 0.07 h; Fig. S5*A*: 6.73 ± 0.08 h), the lag time of fibril formation was significantly decreased in these mutations in the presence of 2% (v/v) preformed seed fibrils of H177R, R177H, N159D, and D159N, respectively (Fig. S6*B*: 10.25 ± 0.06 h, *p* = 0.00026; Fig. S6*D*: 3.48 ± 0.03 h, *p* = 0.0000073; Fig. S6*F*: 10.12 ± 0.34 h, *p* = 0.0016; Fig. S6*F*: 5.65 ± 0.03 h, *p* = 0.00093). In sharp contrast, the addition of 2% preformed seed fibrils of H177R, R177H, N159D, and D159N did not significantly influence the elongation phase of fibril formation of H177R, R177H, N159D, and D159N, respectively (Fig. S6; controls: Figs. 5 and S5). The aforementioned experiments help drive the narrative that the primary effects of the mutations are on nucleation rather than elongation phase of fibril formation of PrP. Moreover, the lag time of H177R fibrillization in the presence of 2% preformed seed fibrils of H177R (Fig. S6*B*: 10.25 ± 0.06 h, *p* = 0.000011) was much significantly higher than that of wildtype human PrP in the presence of 2% preformed seed fibrils of the wildtype human protein (Fig. S6*B*: 4.55 ± 0.03 h), indicating that in the presence of preformed seed fibrils, the H177R mutation delays the kinetics of fibril formation of human PrP more significantly than in the absence of preformed seed fibrils. Similarly, in the presence of preformed seed fibrils, the R177H mutation promotes the kinetics of fibril formation of dog PrP more significantly than in the absence of preformed seed fibrils (Fig. S6*D*). It should be mentioned that the nucleation phase was not completely bypassed by preformed seed fibrils so that the lag phases in comparisons shown in Fig. S6, *A*, *C*, and *E* did not overlap one another.

Altogether, these data strongly suggest that Arg177/Asp159 from the dog PrP and His177/Asn159 from the human PrP control fibril formation *per se*. H177R and N159D inhibit PrP fibrillization, but R177H and D159N promote PrP fibrillization. Importantly, Arg177 and Asp159 from the dog PrP inhibit amyloid formation of the human protein by increasing the lag time of its fibrillization (Figs. 5, S4, and S5) and blocking its structural instability (Fig. S7, *A* and *B*), but His177 and Asn159 from the human PrP promote fibril formation of the canid protein by decreasing the lag time of its fibrillization (Figs. 5, S4, and S5) and triggering its structural instability (Fig. S7, *C* and *D*). Therefore, Arg177/Asp159 from the dog PrP and His177/Asn159 from the human PrP are crucial amino acids in modulating PrP aggregation.

### The influence of glycosylphosphatidylinositol anchoring and N-linked glycosylation on the impacts of Arg177/His177 and Asp159/Asn159

**Figure 7:**
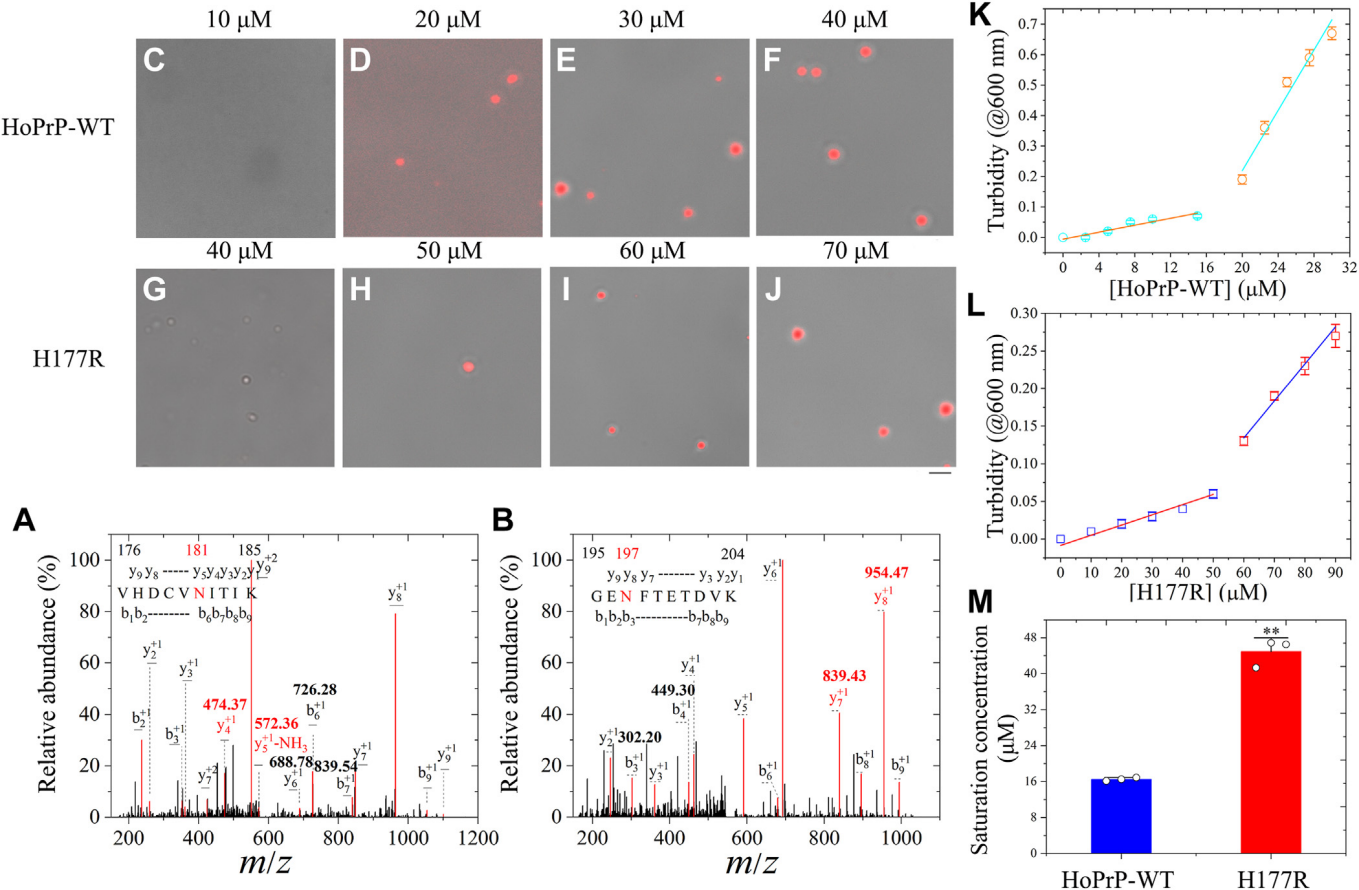
**Arg177 from the dog PrP strongly slows down the LLPS of GPI-anchored and glycosylated full-length human PrP**^**C**^**, shifting the equilibrium phase boundary to a higher protein concentration.** Full-length wildtype human PrP^C^ and its single variant H177R were expressed and purified in sf9 insect cells. *A* and *B*, identification of N-linked glycosylation sites in the insect cell–purified HoPrP-WT using mass spectrometry (MS). The Coomassie blue–stained gels of SDS-PAGE of GPI-anchored and glycosylated HoPrP-WT were scissored out, chopped, trypsinized, and then analyzed with nano-LC–MS/MS. *A*, an MS^2^ analysis of parent peptide V^176^HDCVNITIK^185^ digested by trypsin. Analysis of the y-ions ($y_{5}^{+1}$-NH_3_ and $y_{4}^{+1}$) indicates +1 Da mass shift for an Asn-to-Asp conversion at position 181 (572.36 plus 17.026 minus 474.37 = 115.02 Da, 115.02 minus 114.04 = 0.98 Da), demonstrating N-linked glycosylation at Asn181 in the insect cell–purified HoPrP-WT. *B*, an MS^2^ analysis of parent peptide G^195^ENFTETDVK^204^ digested by trypsin. Analysis of the y-ions ($y_{8}^{+1}$ and $y_{7}^{+1}$) also indicates +1 Da mass shift for an Asn-to-Asp conversion at position 197 (954.47 minus 839.43 = 115.04 Da, 115.04 minus 114.04 = 1.00 Da), demonstrating N-linked glycosylation at Asn197 in the insect cell–purified HoPrP-WT. *C*−*J*, 10 (*C*), 20 (*D*), 30 (*E*), 40 (*F* and *G*), 50 (*H*), 60 (*I*), or 70 (*J*) μM post-translationally modified wildtype human PrP^C^ (HoPrP-WT) (*C*−*F*) and H177R PrP^C^ (*G*−*J*) were labeled by TAMRA (*red fluorescence*) and incubated with 1× PBS (pH 7.4) containing 12.5% (w/v) PEG 8000 on ice to induce LLPS for 10 min. Liquid droplets of the human PrP^C^ were observed by confocal microscopy, with excitation at 561 nm. *C*−*J*, the merged images of the TAMRA images in Fig. S8, *A*−*D* and *I*−*L*, and the brightfield images in Fig. S8, *E*−*H* and *M*−*P*. The scale bar represents 10 μm. *K* and *L*, the turbidity of PrP^C^ condensates was measured at 600 nm and 25 °C. The dependence of turbidity changes for LLPS of GPI-anchored and glycosylated HoPrP-WT (*K*) or its single variant H177R (*L*) on the concentration of HoPrP-WT ([HoPrP-WT]) or H177R ([H177R]) was expressed as mean ± SD (with error bars) of values obtained in three independent experiments. Representative calculation based on turbidity measurements to determine saturation concentration of HoPrP-WT (*open circle*) or H177R (*open square*). The *orange* and *red lines* are drawn through data points indicating the absence of LLPS, whereas the *cyan* and *blue lines* are drawn through data points in which robust LLPS of HoPrP-WT and H177R occurs, respectively. The concentration of protein at which these two lines intersect is an estimation of the saturation concentration. *M*, saturation concentrations of HoPrP-WT (*blue*) and H177R (*red*) (*open black circles* shown in scatter plots) were expressed as the mean ± SD (with error bars) of values obtained in three independent experiments. H177R, *p* = 0.0032. Statistical analyses were performed using the Student's *t* test. Values of *p* < 0.05 indicate statistically significant differences. The following notation is used throughout: ∗*p* < 0.05; ∗∗*p* < 0.01; and ∗∗∗*p* < 0.001 relative to control (the saturation concentration for wildtype PrP^C^). GPI, glycosylphosphatidylinositol; LLPS, liquid–liquid phase separation; PrP, prior protein; PrP^C^, cellular prion protein; TAMRA, 5(6)-carboxy-tetramethylrhodamine *N*-succinimidyl ester.

PrP is a glycosylphosphatidylinositol (GPI)-anchored glycoprotein with two N-linked glycosylation sites, Asn181 and Asn197, in its C-terminal domain (59). We expand on the limitations of the aforementioned study. First, the recombinant PrP molecules we used previously are not glycosylated and lack both these post-translational modifications. Second, the aforementioned study does not consider the influence of GPI anchoring and N-linked glycosylation of PrP on the impacts of Arg177/His177 and Asp159/Asn159. Such an influence could be especially pronounced for the case of residue 177, which is right next to one of the N-linked glycosylation sites (Fig. S1). Therefore, the impact of our studies would be considerably enhanced by testing our basic conclusions in cell cultures, or other experimental systems, that contain the usually GPI-anchored and glycosylated PrP (Figs. 7, S8, and S9). To investigate this, we expressed and purified full-length wildtype human PrP^C^ and its single variant H177R in sf9 insect cells and identified N-linked glycosylation sites in the wildtype using mass spectrometry (MS). Analysis of the y-ions in Figure 7, *A* and *B*, indicated +1 Da mass shift for an Asn-to-Asp conversion at positions 181 and 197, demonstrating N-linked glycosylation at both Asn181 and Asn197 in the insect cell–purified protein. Insect cell–purified wildtype human PrP^C^ and H177R PrP^C^ were labeled by TAMRA (red fluorescence) and incubated with 1× PBS (pH 7.4) containing 12.5% (w/v) PEG 8000 on ice. We did observe the LLPS of GPI-anchored and glycosylated wildtype human PrP^C^ and H177R PrP^C^ by confocal microscopy (Fig. 7, *C*−*J*). In total, 10 μM wildtype human PrP^C^ did not form any liquid droplets, the wildtype at 20 μM did produce a few liquid droplets, and 30 or 40 μM wildtype human PrP^C^ formed abundant liquid droplets in PBS buffer containing 12.5% PEG 8000 on ice (Figs. 7, *C*−*F*, and S8, *A*−*H*). However, 40 μM H177R PrP^C^ did not form any liquid droplets, and 50, 60, or 70 μM H177R PrP^C^ did produce a few liquid droplets in the same buffer on ice (Figs. 7, *G*−*J*, and S8, *I*−*P*). The TAMRA images (Fig. S8, *A*−*D* and *I*−*L*) and the brightfield images (Fig. S8, *E*−*H* and *M*−*P*) are of the same field of view and are mostly overlapping proved by the merged images (Fig. 7, *C*−*J*) for the same experiments collected at the same focal plane. To assess the effect of Arg177 from the dog PrP on GPI-anchored and glycosylated human PrP^C^ propensity for LLPS, we determined saturation concentrations of the wildtype and the single variant by measuring the turbidity of PrP^C^ condensates at 600 nm as a function of the concentration of PrP^C^ (Fig. 7, *K* and *L*). These data clearly demonstrate that the saturation concentration was 16.53 ± 0.38 μM for wildtype human PrP^C^ and 44.9 ± 3.1 μM for its single variant H177R (Fig. 7*M*). Importantly, we found that the saturation concentration of the insect cell–purified H177R (*p* = 0.0032) was significantly higher than that of the insect cell–purified wildtype human PrP^C^ (Fig. 7*M*). Together, the data showed that Arg177 from the dog PrP also strongly slows down the LLPS of GPI-anchored and glycosylated full-length human PrP^C^, shifting the equilibrium phase boundary to a higher protein concentration.

We have shown that Arg177 and Asp159 from the dog PrP inhibit amyloid formation of the human protein. We wanted to know whether these two residues could suppress the aggregation of human PrP in cell cultures. We detected insoluble PrP aggregates in the sarkosyl-insoluble ultracentrifugation pellets using the anti-PrP monoclonal antibody 3F4 (Fig. S9, *A* and *B*). Human embryonic kidney 293T (HEK-293T) cells stably expressing full-length wildtype human PrP^C^, H177R PrP^C^, or N159D PrP^C^ and Madin–Darby canine kidney (MDCK) cells stably expressing full-length wildtype dog PrP^C^, R177H PrP^C^, or D159N PrP^C^ formed PrP aggregates when cultured for 2 days (Fig. S9, *A* and *B*). We found that ∼64% of total human PrP ended up in the pellet when the cells overexpressing the wildtype human protein were cultured for 2 days. There is a decrease in pellet size when the cells overexpressing H177R or N159D were cultured for 2 days (∼22% or ∼43%, Fig. S9*A*). We also found that ∼30% of total dog PrP ended up in the pellet when the cells overexpressing the wildtype dog protein were cultured for 2 days. There is an increase in pellet size when the cells overexpressing R177H or D159N were cultured for 2 days (∼90% or ∼50%, Fig. S9*B*). Notably, the normalized amount of H177R PrP aggregates or N159D PrP aggregates in the detergent-insoluble pellets was significantly lower than that in the control pellets (*p* = 0.00018 or 0.014) (Fig. S9*C*), indicating that the cells overexpressing H177R or N159D produced much fewer PrP aggregates than those overexpressing the wildtype human protein. In sharp contrast, the normalized amount of R177H PrP aggregates or D159N PrP aggregates in the detergent-insoluble pellets was significantly higher than that in the control pellets (*p* = 0.00085 or 0.012) (Fig. S9*D*), indicating that the cells overexpressing R177H or D159N produced much more PrP aggregates than those overexpressing the wildtype dog protein. Our immunoblotting data demonstrated that Arg177 and Asp159 from the dog PrP significantly inhibited the aggregation of human PrP in cell cultures, but His177 and Asn159 from the human PrP greatly enhanced the aggregation of dog PrP in cell cultures.

Together, these results confirm our basic conclusions in cell cultures, or other experimental systems, that contain the GPI-anchored and glycosylated PrP.

## Discussion

Recently, several *in vitro* and *in vivo* studies have demonstrated that Asp159 from the dog PrP plays a critical role in encoding higher structural stability of PrP in prion-resistant canids (26, 27, 28, 29, 30, 33, 34, 35). Because Arg177 and Asp159 cause unique charge distribution patterns on the front and back sides of dog PrP^C^, it has generally been thought that these two unique amino acid residues might be responsible for the prion resistance of canids (27, 28, 29, 52, 53). Accumulating pieces of evidence point to a crucial role of the amino acid residue at position 159 (Asp159 or Glu159) of dog PrP^C^ in preventing PrP conformational change and disease (26, 27, 28, 29, 30), whereas the amino acid residue substitution D159N is highly toxic (26) and is responsible for the recovered susceptibility to PrP misfolding (29). We now show that Arg177 and Asp159 from the dog PrP strongly inhibit amyloid formation of human PrP by increasing the lag time of its fibrillization and blocking its structural instability, but His177 and Asn159 from the human PrP greatly promote fibril formation of dog PrP by decreasing the lag time of its fibrillization and triggering its structural instability. Moreover, Arg177 and Asp159 from the dog PrP strongly inhibit the aggregation of human PrP in cell cultures, but His177 and Asn159 from the human PrP greatly promote the aggregation of dog PrP in cell cultures. Strikingly, the enhancing effect or inhibiting effect of a single amino acid substitution D159N or N159D on PrP fibril formation is remarkably smaller than R177H or H177R. Interestingly, a single amino acid substitution G26R greatly promotes fibril formation of human apolipoprotein A-I by decreasing the lag time of its fibrillization, but another amino acid residue substitution G26E strongly inhibits its fibril-forming propensity (60).

In this work, we report that PrP^C^, a GPI-anchored glycoprotein existing in membrane-bound and cytoplasmic forms (59, 61), exhibits disparate propensities to phase separate in cellular crowded environments, and that Arg177 and Asp159 from the dog PrP and His177 and Asn159 from the human PrP are key amino acid residues in modulating PrP^C^ liquid-phase condensation. We show that Arg177 and Asp159 from the dog PrP slow down the LLPS of full-length human PrP^C^ by increasing the saturation concentration for PrP condensation dramatically, but His177 and Asn159 from the human PrP promote the LLPS of full-length dog PrP^C^ by decreasing the saturation concentration for PrP condensation remarkably. It should be mentioned that the enhancing or inhibiting effect of a single amino acid substitution D159N or N159D on the LLPS of PrP^C^ are remarkably smaller than R177H or H177R. According to Figures 3 and 4, H177R and N159D enhance but R177H and D159N reduce fluorescence recovery. This means that H177R and N159D increase but R177H and D159N decrease the fluidity of LLPS condensates, possibly because these mutations, carrying an amino acid switch from His to Arg at residue 177 or a switch from Asn to Asp at residue 159, could modulate the formation of some other kind of solid with low mobility from phase-separated PrP^C^ condensates. Unfortunately, we have not documented fibril formation from the condensates generated in the absence of GdnHCl as was done in Figure 1, Figure 2, Figure 3, Figure 4. Importantly, we find that the H177R and N159D mutations help maintain liquid-like properties of PrP^C^ condensates and protein solubility, which are disrupted in the presence of the R177H and D159N mutations. Overall, our results show that the R177H and D159N mutations promote the LLPS of PrP^C^ and the highly condensed assembly, rendering the resulting liquid droplets solid like. In sharp contrast, the H177R and N159D mutations slow down the LLPS of PrP^C^ and the highly condensed assembly, maintaining liquid-like properties of the condensates. Thus, the LLPS of PrP^C^ is negatively modulated by the H177R and N159D mutations but positively modulated by the R177H and D159N mutations. The fact that the phase separation and aggregation of PrP are delayed by the H177R and N159D mutations but are accelerated by the R177H and D159N mutations highlights the significance of phase transition pathways in the functional and pathological aspects of PrP. Intriguingly, the LLPS of PrP^C^ is positively modulated by the Y145Stop mutation (47) that is associated with a familial prion disease (62) but negatively modulated by a protective mutation G127V (44). Strikingly, LLPS of the prion-like proteins FUS, TDP-43, and TIA1 is also positively modulated by disease-causing mutations (63, 64, 65).

PrP^C^ contains a low-complexity IDR in its largely disordered N-terminal domain (residues 23−120) and a folded C-terminal globular domain (residues 121−231) (7, 45, 47, 50, 66). It is thought that IDRs often encode driving forces for the LLPS of PrP^C^ (42, 46, 47, 49, 50, 51) and prion-like proteins (67). In this study, we surprisingly observed that Arg177/His177 and Asp159/Asn159 in the C-terminal domain are key residues in modulating liquid-phase condensation of full-length PrP^C^. In addition, our results show that Arg177/His177 and Asp159/Asn159 in the C-terminal domain slow/enhance the LLPS of full-length human/dog PrP^C^, shifting the equilibrium phase boundary to higher/lower protein concentrations and inhibit/promote amyloid fibril formation of the human/canid protein. Overall, this study presents some interesting data elucidating the potential role of His177 and Asn159 in the C-terminal domain in driving the LLPS of PrP^C^. Previously, it had been proposed that it was the disordered N-terminal domain of PrP that drove LLPS (42, 46, 47, 49, 50, 51), but the findings presented here clearly show a role for these two C-terminal residues. Therefore, the LLPS of PrP^C^ is driven by an IDR in its unstructured N-terminal domain and is regulated by key amino acids in its C-terminal globular domain.

In order to decipher how the H177R and N159D mutations, which carry an amino acid switch from His to Arg at residue 177 or a switch from Asn to Asp at residue 159, impact the LLPS of PrP^C^, we employed a stickers-and-spacers model for LLPS of proteins with disordered prion-like domains (68). Because Arg has the strongest positive charge among the three basic amino acids, Arg, Lys, and His, the H177R and N159D mutations increase the charge content in PrP^C^, which destabilize the LLPS of PrP^C^ possibly by increasing the effective solvation volumes and electrostatic repulsions (68).

**Figure 8:**
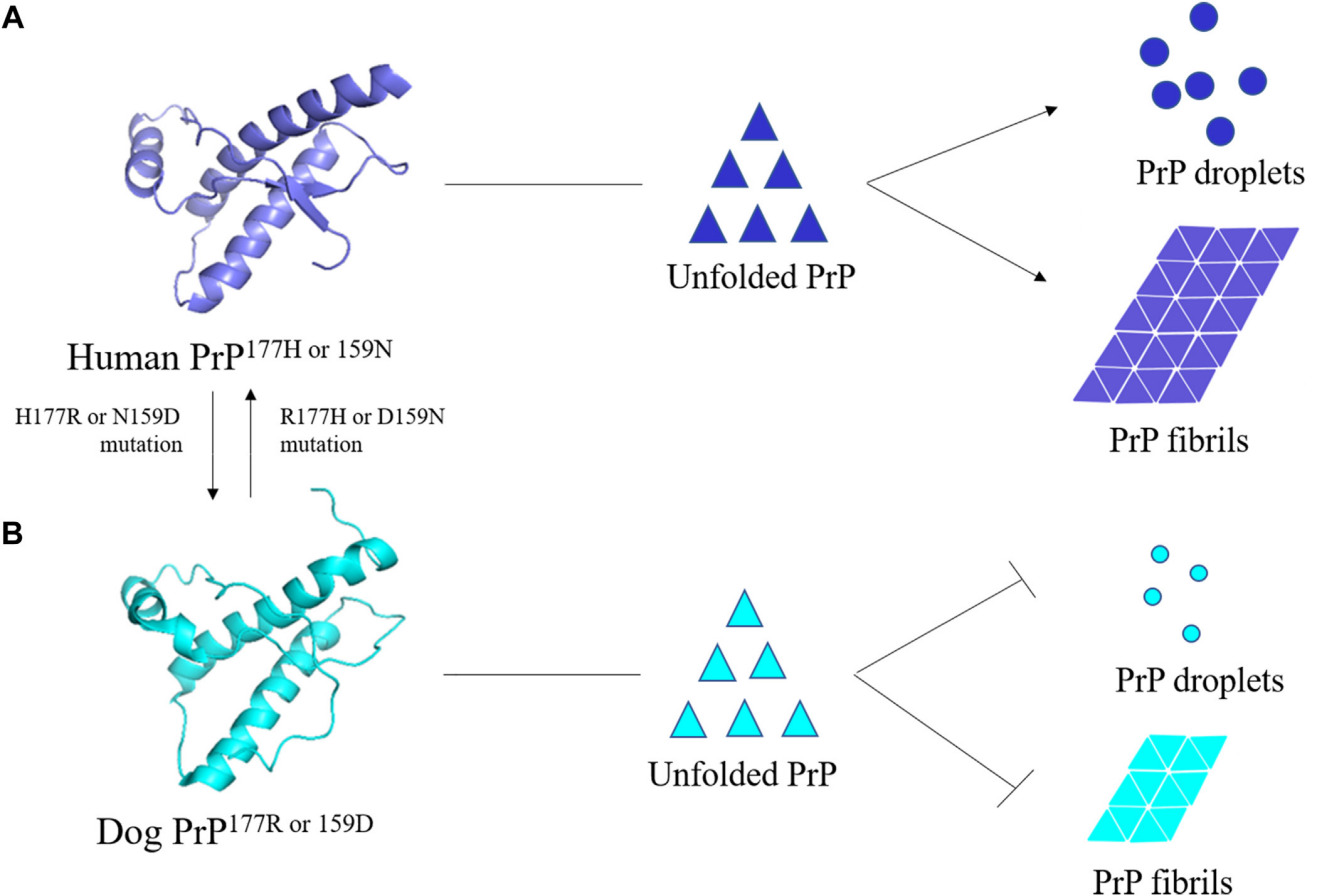
**Arg177 and Asp159 from the dog PrP slow LLPS and inhibit amyloid formation of human PrP.** From *B* to *A*, a hypothetical model shows how a single residue His177 or Asn159 enhances LLPS (more PrP^C^ droplets, *blue balls*, are produced) and promotes fibril formation (more PrP fibrils, *blue bricks*, are formed) of PrP. *A*, ribbon representation (*blue*) of the structure of the C-terminal domain of human PrP^C^ (*A*, *left*) (Protein Data Bank code: 1QLX) (66). Unfolded PrP, *blue triangles* (*A*, *middle*). From *A* to *B*, a hypothetical model shows how a single residue Arg177 or Asp159 slows down LLPS (less PrP^C^ droplets, *green balls*, are produced) and inhibits amyloid formation (less PrP fibrils, *green bricks*, are formed) of PrP. *B*, ribbon representation (*green*) of the structure of the C-terminal domain of dog PrP^C^ (*A*, *left*) (Protein Data Bank code: 1XYK) (52). Unfolded PrP, *green triangles* (*A*, *middle*). LLPS, liquid–liquid phase separation; PrP, prior protein; PrP^C^, cellular prion protein.

In summary, our results describe a model to underpin molecular hypotheses of how Arg177 and Asp159 in the C-terminal domain of the dog PrP slow down the LLPS of PrP^C^ and inhibit amyloid formation of PrP implicated in prion disease resistance in dogs (Fig. 8). We describe influences of the two key residues that, in dog PrP, reduce its tendency to undergo LLPS and amyloid fibril formation. Importantly, we show that a single residue His177 or Asn159 from the human PrP enhances the LLPS of full-length dog PrP^C^ and promotes amyloid fibril formation of the canid protein; more PrP^C^ droplets and more PrP fibrils are produced (Fig. 8). A single residue Arg177 or Asp159 from the dog PrP, however, slows down the LLPS of full-length human PrP^C^ and inhibits amyloid formation of the human protein; less PrP^C^ droplets and less PrP fibrils are formed (Fig. 8). We thus suggest that the formation of PrP^C^ droplets and PrP fibrils are separate pathways under the conditions of this article. These results are of interest to the prion field in determining the underpinnings of why dogs are more resistant to prion diseases than humans and other mammalian species. Our study enhances our understanding of how LLPS and abnormal aggregation of PrP are inhibited by naturally occurring sequence features. The selective regulation of mutations carrying an amino acid switch from His to Arg at residue 177 or a switch from Asn to Asp at residue 159 on the formation of some other kind of solid with low mobility from phase-separated PrP^C^ condensates will be valuable with regard to understanding the functional basis underlying LLPS of proteins and inspiring future research on protein condensation diseases caused by abnormal liquid- or solid-like states of proteins (69) and positively regulated by disease-causing mutations (47, 63, 64, 65) or negatively regulated by protective mutations (42, 44).

## Experimental procedures

### Materials

Two dyes, ThT and ANS, and mouse anti-β-actin antibody (A1978) were purchased from Sigma–Aldrich. The mouse anti-PrP monoclonal antibody 3F4 (catalog no.: 800307) was obtained from BioLegend. GdnHCl was obtained from Promega. Sarkosyl was purchased from Amresco. Ni-Sepharose was purchased from GE Company. All other chemicals used in this study were of analytical grade and were produced in China.

### Protein purification

A plasmid-encoding full-length human PrP (residues 23−231) was a gift from Dr Geng-Fu Xiao (Wuhan Institute of Virology, Chinese Academy of Sciences), and a plasmid-encoding full-length dog PrP (residues 23−231) was a gift from Dr Adriano Aguzzi (University of Zurich). The genes for human PrP 23−231 and dog PrP 23−231 were constructed in the vector pET-30a (+), and PrP mutants H177R, N159D, R177H, and D159N were constructed by site-directed mutagenesis using a wildtype PrP template; the primers are shown in Table S1. All PrP plasmids were transformed into *Escherichia coli*. Recombinant full-length wildtype human PrP and its variants H177R and N159D, as well as recombinant full-length wildtype dog PrP and its variants R177H and D159N, were expressed in *E. coli* BL21 (DE3) cells (Novagen, Merck) and purified by high-performance liquid chromatography on a C4 reverse-phase column (Shimadzu) as described by Bocharova *et al.* (70) and Zhou *et al.* (19). After purification, recombinant wildtype human PrP^C^, H177R PrP^C^, N159D PrP^C^, wildtype dog PrP^C^, R177H PrP^C^, and D159N PrP^C^ were dialyzed against 1× PBS (pH 7.4) for 24 h, concentrated, filtered, and stored at −80 °C. The target genes for human PrP 23−231 and its mutant H177R were subcloned into the pFastBac vector (Thermo Fisher Scientific) by double digestion technology, and the two plasmids were separately transformed into the DH10Bac strains (Gibco). Recombinant Bacmid plasmids were transfected into sf9 insect cells. After 48 h of transfection, the medium was collected and centrifuged at 5000*g* for 5 min at 4 °C to remove the cell debris. The supernatant obtained was the first generation of baculovirus (P1). A first batch of virus P1 was used to reinfect sf9 insect cells for 48 h to produce the second generation of baculovirus (P2). For large-scale expression, sf9 insect cells were infected with the P2 batch of virus for 48 h. The infected sf9 insect cells were collected and centrifuged at 10,000*g* for 15 min at 4 °C to remove the cell debris. The supernatant was sonicated at 250 W for 30 min and filtered through a 0.45 μm filter (Whatman). The recombinant full-length wildtype human PrP and its variant H177R were purified by the method of size-exclusion chromatography using an ÄKTA Pure instrument (GE Healthcare). After purification, recombinant wildtype human PrP^C^ and H177R PrP^C^ were dialyzed against 1× PBS (pH 7.4) for 24 h and concentrated. SDS-PAGE and MS were used to confirm that the purified human PrP proteins and the purified dog PrP proteins were single species with an intact disulfide bond. We used a NanoDrop OneC Microvolume UV-Vis Spectrophotometer (Thermo Fisher Scientific) to determine the concentrations of wildtype human PrP^C^ and its variants H177R and N159D PrP^C^, as well as wildtype dog PrP^C^ and its variants R177H and D159N PrP^C^, using their absorbances at 280 nm and the molar extinction coefficients calculated from the composition of the proteins (http://web.expasy.org/protparam/).

### Liquid-droplet formation

The freshly bacterial-purified wildtype human PrP^C^ and its variants H177R and N159D PrP^C^, wildtype dog PrP^C^ and its variants R177H and D159N PrP^C^, as well as the freshly insect cell–purified wildtype human PrP^C^ and its variant H177R were incubated with TAMRA (red fluorescence, excitation at 561 nm) at a PrP^C^:TAMRA molar ratio of 1:3 for 1 h. These labeled proteins were filtered, concentrated to 200 μM in a centrifugal filter (Millipore), and diluted in 1× PBS (pH 7.4). In total, 50 μM wildtype human PrP^C^ and its variant H177R PrP^C^ labeled by TAMRA as well as wildtype dog PrP^C^ and its variant R177H PrP^C^ labeled by TAMRA were incubated with 1× PBS (pH 7.4) containing 10% (w/v) PEG 8000 on ice to induce LLPS for 10 min. In total, 5, 10, 20, and 40 μM wildtype human PrP^C^ and its variants H177R and N159D PrP^C^ labeled by TAMRA as well as wildtype dog PrP^C^ and its variants R177H and D159N PrP^C^ labeled by TAMRA were incubated with PBS buffer containing 10% PEG 8000 on ice to induce LLPS for 10 min. In total, 10, 20, 30, 40, 50, 60, and 70 μM post-translationally modified wildtype human PrP^C^ and its variant H177R labeled by TAMRA were incubated with 1× PBS (pH 7.4) containing 12.5% (w/v) PEG 8000 on ice to induce LLPS for 10 min. Liquid droplets of PrP^C^ (protein condensates) formed under such conditions were observed by differential interference contrast confocal microscopy using a Leica TCS SP8 laser scanning confocal microscope (Wetzlar) with excitation at 561 nm. All phase separation experiments were performed at least three times and were pretty reproducible.

### Turbidity assays

The turbidity of PrP^C^ condensates was measured at 600 nm and 25 °C using a Cytation 3 Cell Imaging Multi-Mode Reader (BioTek). All turbidity assays were repeated at least three times and were pretty reproducible.

### FRAP

In total, 50 μM wildtype human PrP^C^ and its variants H177R and N159D PrP^C^ labeled by TAMRA as well as wildtype dog PrP^C^ and its variants R177H and D159N PrP^C^ labeled by TAMRA were incubated with PBS buffer containing 10% PEG 8000 on ice to induce LLPS for 10 min. Liquid droplets of human/dog PrP^C^ were observed by a Leica TCS SP8 laser scanning confocal microscope with excitation at 561 nm. For each droplet, a square was bleached at 80% transmission for 2 s, and postbleaching, time-lapse images were collected (30 frames, 5 s per frame). Images were analyzed using Zen (LSM 880 confocal microscope manufacturer’s software). All FRAP experiments were repeated three times, and the results were reproducible.

### PrP fibril formation

The freshly bacterial-purified wildtype human PrP^C^ and its variants H177R and N159D PrP^C^ as well as wildtype dog PrP^C^ and its variants R177H and D159N PrP^C^ (20 μM) were incubated in 1× PBS (pH 7.4) containing 2 M GdnHCl with agitation at 220 rpm at 37 °C for 6 to 12 h, 18 to 30 h, and 6 to 18 h, as well as 8 to 16 h, 4 to 8 h, and 4 to 10 h, respectively, and the wildtype human/dog PrP, H177R, N159D, R177H, and D159N fibrils were collected for TEM experiments.

### ThT binding assays

The freshly bacterial-purified wildtype human PrP^C^ and its variants H177R and N159D PrP^C^ as well as wildtype dog PrP^C^ and its variants R177H and D159N PrP^C^ (20 μM) were incubated in PBS buffer containing 2 M GdnHCl with agitation at 220 rpm at 37 °C for 13, 30, and 24 h as well as 18, 14, and 14 h, respectively, and then analyzed by ThT binding assay. The freshly bacterial-purified wildtype human PrP^C^, its variant H177R PrP^C^, wildtype dog PrP^C^, and its variant R177H PrP^C^ (10 μM) were incubated in 1× PBS (pH 7.4) at 25 and 53 °C for 1 h, respectively, with agitation at 220 rpm, and then analyzed by ThT binding assay. The final concentrations of human/dog PrP and ThT were 1 and 125 μM, respectively. A Cytation 3 Cell Imaging Multi-Mode Reader was used to measure ThT fluorescence produced, with excitation at 450 nm and emission at 480 nm. The lag time of the fibrillization of wildtype human/dog PrP and its mutants was determined using a sigmoidal equation (44, 71, 72) using the ThT fluorescence data. Statistical analyses were performed using the Student's *t* test. The following notation is used throughout: ∗*p* < 0.05; ∗∗*p* < 0.01; ∗∗∗*p* < 0.001; and ∗∗∗∗*p* < 0.0001 relative to wildtype PrP.

### ANS binding assays

The freshly bacterial-purified wildtype human PrP^C^, its variant H177R PrP^C^, wildtype dog PrP^C^, and its variant R177H PrP^C^ (20 μM) were incubated in PBS buffer containing 2 M GdnHCl with agitation at 220 rpm at 37 °C for 13, 30, 18, and 14 h, respectively, or incubated in 1× PBS (pH 7.4) at 25 and 53 °C for 1 h, respectively, with agitation at 220 rpm, and then analyzed by ANS binding assay. The final concentrations of human/dog PrP and ANS were 1 and 125 μM, respectively. A Cytation 3 Cell Imaging Multi-Mode Reader was used to measure the fluorescence spectra for ANS binding recorded between 400 and 700 nm, with excitation at 385 nm. The lag time of the fibrillization of wildtype human/dog PrP and its single variant was determined using a sigmoidal equation (44, 71, 72) using the ANS fluorescence data.

### SDS-PAGE of sarkosyl-soluble PrP

To distinguish human/dog PrP monomers, which are sarkosyl soluble, from human/dog PrP fibrils, which are sarkosyl insoluble, sarkosyl-soluble SDS-PAGE experiments for fibril formation of wildtype human PrP^C^ and its variants H177R and N159D PrP^C^ as well as wildtype dog PrP^C^ and its variants R177H and D159N PrP^C^ were carried out as previously described in detail (19). In total, 20 μM PrP samples were taken at multiple time points during aggregation, incubated with 2% sarkosyl for 0.5 h at 25 °C, then mixed with the 2× loading buffer (without β-mercaptoethanol and heating), and separated by 15% SDS-PAGE. The soluble human/dog PrP monomers were detected by SDS-PAGE with Coomassie Blue R250 staining.

### TEM of PrP fibrils

Human/dog PrP fibrils were examined by TEM of negatively stained samples. About 10 μl of PrP fibril samples (20 μM) were taken at multiple time points during aggregation, loaded on copper grids for 60 s, and washed with water for 10 s. Samples on grids were then stained with 2% (w/v) uranyl acetate for 30 s and dried in air at 25 °C. The stained samples were examined using a Talos L120C G2 transmission electron microscope operated at 120 kV (Thermo Fisher Scientific).

### Cryo-EM of PrP fibrils

Amyloid fibrils of human/dog PrP were produced as previously described in detail (7, 8). An aliquot of 3.5 μl of ∼13 μM PrP fibril solution was applied to glow-discharged holey carbon grids (Quantifoil Cu R1.2/1.3, 300 mesh), blotted for 3 s, and plunge-frozen in liquid ethane using a Vitrobot Mark IV. The cryo-EM micrographs were acquired on a Glacios transmission electron microscope operated at 200 kV and equipped with a field emission gun and a Ceta-D CMOS camera (Thermo Fisher Scientific).

### Seeding experiments

Mature amyloid fibrils of human/dog PrP were sonicated on ice using a probe sonicator for 30 s (interval of 5 s, 200 W) in order to produce the seeds used in the seeding experiments. The kinetics of fibril formation of samples with 2% (v/v) preformed seed fibrils at the initial time were monitored by ThT binding assays.

### Nano-LC–MS/MS analysis

Full-length wildtype human PrP^C^ and its single variant H177R were expressed and purified in sf9 insect cells. The Coomassie blue–stained gels of SDS-PAGE of GPI-anchored and glycosylated wildtype human PrP^C^ were scissored out, chopped, digested by trypsin, and then analyzed by nano-LC on an LTQ Orbitrap XL mass spectrometer (Thermo Fisher Scientific). About 60 μg of peptide mixtures was pressure-loaded onto a 12 cm silica capillary column with filter packed with 10 cm PS/DVB polymer resin (5 μm Hydro-RP 800 Å; Welch). Such a polymer resin silica capillary column (100 μm id) was used as analysis column. The buffer solutions used were 0.1% formic acid in water (buffer A) and 0.1% formic acid in acetonitrile (buffer B). The column was first desalted with buffer A and then eluted by a nonlinear increasing gradient of buffers A to B during 50 min at 800 nl/min flow rate. The gradient contained 5 min of 5% buffer B, 35 min of 25% buffer B, 5 min of 90% buffer B, and finally 5 min of 5% buffer B. The LTQ-Orbitrap was operated in Fourier transform MS profile full MS mode with the Xcalibur software (Thermo Fisher Scientific). Nano-electrospray ionization was accomplished with a spray voltage of 2.1 kV and a heated capillary temperature of 180 ^°^C. Injection time was set to 500 ms, and two microscans were set in the Orbitrap in the 500 to 1700 *m/z* range with the resolution set to a value of 100,000.

### Cell culture and transfection

HEK-293T cells (catalog number: GDC0187) and MDCK cells (catalog number: GDC0012) were obtained from China Center for Type Culture Collection and cultured in Dulbecco’s modified Eagle’s medium and in minimum essential media (Gibco, Invitrogen), respectively, supplemented with 10% (v/v) fetal bovine serum (Gibco), 100 U/ml streptomycin, and 100 U/ml penicillin in 5% CO_2_ at 37 °C. HEK-293T cell line stably expressing full-length wildtype human PrP^C^, H177R PrP^C^, or N159D PrP^C^ and MDCK cells stably expressing full-length wildtype dog PrP^C^, R177H PrP^C^, or D159N PrP^C^ were constructed with a lentiviral vector construction system (pHAGE-puro). The target DNA fragments were inserted into the lentiviral vector, and the plasmids containing target DNA, pVSVG, and p976 were packaged in HEK-293T cells at a ratio of 2:1:1 by Lipofectamine 2000 (Invitrogen). The ratio of liposome to DNA was 2:1. After 48 h of transfection, the viruses were harvested and filtered, and then HEK-293T and MDCK cells were infected with the packaged lentivirus twice for 12 h each with a 12 h interval. In order to establish the stable cell lines, puromycin was used to screen overexpressed cells. The expression of each protein was detected by Western blot.

### Sarkosyl-insoluble Western blotting

Sarkosyl-insoluble Western blotting was used to investigate intracellular PrP aggregates. Cell lysates from the aforementioned HEK-293T stable cells and MDCK stable cells, both cultured for 2 days, were centrifuged at 17,000*g* for 30 min at 4 °C to remove the cell debris. Half of the supernatant was incubated with 1% sarkosyl for 30 min at 25 °C. The mixture was then ultracentrifuged at 150,000*g* for 30 min, and the pellets were washed twice with 1× PBS (pH 7.4). The sarkosyl-insoluble pellets were boiled in the SDS-PAGE loading buffer for 10 min. The other half of the supernatant, which served as the total protein sample, was also boiled in the SDS-PAGE loading buffer for 10 min. The samples were separated by 12.5% SDS-PAGE and then Western blotted as previously described in detail (57). The sarkosyl-insoluble pellets from those cells were probed using the anti-PrP monoclonal antibody 3F4, and the corresponding cell lysates were probed using 3F4 and anti-β-actin antibody, respectively. The amount of loaded protein was normalized using a BCA Protein Quantification kit (Beyotime). For calculating the amounts of sarkosyl-insoluble PrP, the ImageJ software (National Institutes of Health) was used to assess the densitometry of PrP bands. The normalized amount of insoluble PrP aggregates in HEK-293T cells overexpressing human PrP^C^ or MDCK cells overexpressing dog PrP^C^ was determined as a ratio of the density of insoluble PrP aggregate bands over that of the total PrP bands in cell lysates. HEK-293T cells overexpressing wildtype human PrP^C^ or MDCK cells overexpressing wildtype dog PrP^C^ were used as a control. Statistical analyses were performed using the Student's *t* test. Values of *p* < 0.05 indicate statistically significant differences. The following notation is used throughout: ∗*p* < 0.05; ∗∗*p* < 0.01; and ∗∗∗*p* < 0.001 relative to control.

### Statistical analysis

The data shown for each experiment were based on at least three technical replicates, as indicated in individual figure legends. Data are presented as mean ± SD, and *p* values were determined using the Student’s *t* test. All experiments were further confirmed by biological repeats.

## Data availability

All data generated or analyzed during this study are included in this article or available from the corresponding author upon request.

## Supporting information

This article contains supporting information.

## Conflict of interest

The authors declare that they have no conflicts of interest with the contents of this article.
