# Supplementary material for: Arg177 and Asp159 from dog prion protein slow liquid–liquid phase separation and inhibit amyloid formation of human prion protein
Source: J Biol Chem. 2023 Oct 6;299(11):105329. doi: 10.1016/j.jbc.2023.105329 (PMC10641668; doi:10.1016/j.jbc.2023.105329)

**Arg177 and Asp159 from dog prion protein slow liquid–liquid phase separation** **and inhibit** **amyloid formation of human prion protein**

**Xiang-Ning Li^1^,**^‡^**, Yuan Gao^1^,**^‡^**, Yang Li^1^, Jin-Xu Yin^1^, Chuan-Wei Yi^1^, Han-Ye Yuan^1^, Jun-Jie Huang^1^, Li-Qiang Wang^1,2^, Jie Chen^1^, and Yi Liang^1,2^,***

From the ^1^Hubei Key Laboratory of Cell Homeostasis, College of Life Sciences, TaiKang Center for Life and Medical Sciences, Wuhan University, Wuhan 430072, China; ^2^Wuhan University Shenzhen Research Institute, Shenzhen 518057, China

Running Title: Arg177 and Asp159 suppress PrP^C^ phase separation

^‡^ These authors contributed equally to this work.

* For correspondence: Yi Liang, liangyi@whu.edu.cn.

**Keywords:** Prion protein, Protein aggregation, Protein liquid-liquid phase separation, Dog prion protein, Prion diseases, Prion disease resistance

**Supplemental Data**

**Table S1. The primers designed for full-length human PrP with H177R or N159D mutation and full-length dog PrP with R177H or D159N mutation.**

| S-H177R | 5’CAACTTTGTGCGCGACTGCGTCAATATC 3’ |
| --- | --- |
| A-H177R | 5’GATATTGACGCAGTCGCGCACAAAGTTG 3’ |
| S-R177H | 5’ACAACTTTGTGCATGACTGCGTCAACAT 3’ |
| A-R177H | 5’ATGTTGACGCAGTCATGCACAAAGTTGT 3’ |
| S-N159D | 5’GCTACCCAGACCAAGTGTACTACC3’ |
| A-N159D | 5’GGTAGTACACTTGGTCTGGGTAGC3’ |
| S-D159N | 5’CGCTACCCAAACCAAGTGTACTACC3’ |
| A-D159N | 5’GGTAGTACACTTGGTTTGGGTAGCG3’ |

**Supplemental Data**

**Figure S1.** **Sequence alignments of human PrP, mouse PrP, and dog PrP.** The numbering corresponds to the human PrP. Compared with PrP from humans and mice, PrP from dogs has two unique amino acid residues, Arg177 and Asp159 (*red boxes*).

**Figure S2.** **Arg177 from the dog PrP strongly slows down the LLPS of full-length human PrP^C^, but His177 from the human PrP greatly enhances the LLPS of full-length dog PrP^C^.** 5 (*A*, *E*, *I*, and *M*), 10 (*B*, *F*, *J*, and *N*), 20 (*C*, *G*, *K*, and *O*) or 40 (*D*, *H*, *L*, and *P*) μM bacterial purified wild-type human PrP^C^ (HoPrP-WT) (*A*−*D*) and its single variant H177R (*E*−*H*) as well as bacterial purified wild-type dog PrP^C^ (CaPrP-WT) (*I*−*L*) and its single variant R177H (*M*−*P*) were labeled by TAMRA (*red fluorescence*) (*A*−*P*) and incubated with 1 × PBS (pH 7.4) containing 10% (w/v) PEG 8000 on ice to induce LLPS for 10 min. Liquid droplets of the human PrP^C^ or the dog PrP^C^ were observed by DIC confocal microscopy, with excitation at 561 nm. *A*−*P*, TAMRA images. We have replaced panel *C* with a correct version of HoPrP-WT, in which panel *C* in Figs. 2 and S2 does match. The scale bar represents 10 μm.

**Figure S3. Arg177 from the dog PrP strongly slows down the LLPS of full-length human PrP^C^, but His177 from the human PrP greatly enhances the LLPS of full-length dog PrP^C^.** 5 (*A*, *E*, *I*, and *M*), 10 (*B*, *F*, *J*, and *N*), 20 (*C*, *G*, *K*, and *O*) or 40 (*D*, *H*, *L*, and *P*) μM bacterial purified wild-type human PrP^C^ (HoPrP-WT) (*A*−*D*) and its single variant H177R (*E*−*H*) as well as bacterial purified wild-type dog PrP^C^ (CaPrP-WT) (*I*−*L*) and its single variant R177H (*M*−*P*) were labeled by TAMRA (*red fluorescence*) and incubated with 1 × PBS (pH 7.4) containing 10% (w/v) PEG 8000 on ice to induce LLPS for 10 min. Liquid droplets of the human PrP^C^ or the dog PrP^C^ were observed by DIC confocal microscopy, with excitation at 561 nm. *A*−*P*, DIC microscopic images. The scale bar represents 10 μm.

**Figure S4. Arg177 and Asp159 from the dog PrP significantly inhibit amyloid formation of human PrP, but His177 and Asn159 from the human PrP greatly enhance fibril formation of dog PrP.** *A*, SDS-PAGE analysis of time-dependent sarkosyl-soluble PrP, including HoPrP-WT, H177R, N159D, CaPrP-WT, R177H, and D159N. In brief, 20-μM PrP samples were incubated with 2% sarkosyl and separated by 15% SDS-PAGE. The soluble PrP monomers were detected by SDS-PAGE with Coomassie Blue R250 staining. All SDS-PAGE experiments were repeated three times and the results were reproducible. *B* and *C* represent two of the biological replicates of *A*. Marks at the left of the gels indicate the positions of the molecular weight markers.

**Figure S5. Asp159 from the dog PrP strongly inhibits amyloid formation of human PrP, but Asn159 from the human PrP greatly enhances fibril formation of dog PrP.** *A*, Samples (20 μM) of N159D of human PrP (*red*) and D159N of dog PrP (*blue*) were incubated in 1 × PBS (pH 7.4) containing 2 M guanidine hydrochloride (GdnHCl) with agitation at 220 rpm and then analyzed by ThT binding assay. The ThT fluorescence intensity was expressed as the mean ± S.D. (with error bars) of values obtained in three independent experiments. The solid lines show the best exponential fit for the ThT intensity-time curves. The lag time was determined by fitting ThT fluorescence intensity versus time to a sigmoidal equation. Samples (20 μM) of bacterial purified single variants N159D of human PrP (*B*−*D* and *H*) and D159N of dog PrP (*E*−*G* and *I*) were incubated in 1 × PBS (pH 7.4) containing 2 M GdnHCl incubated for 4 (*E*), 6 (*B* and *F*), 10 (*C*, *G*, and *I*), and 18 (*D* and *H*) h with agitation at 220 rpm. *B*−*G*, a 2% (w/v) uranyl acetate solution was used for staining the fibrils negatively. Scale bar: 100 nm. *H* and *I*, cryo-EM micrographs of amyloid fibrils of N159D (*H*) and D159N (*I*). Scale bar: 40 nm.

**Figure S6. The primary effects of the mutations are on the nucleation but not the elongation phase of fibril formation of PrP.** Samples (20 μM) of HoPrP-WT (*blue*), its single variant H177R (*red*) (*A*), CaPrP-WT (*blue*), its single variant R177H (*red*) (*C*), N159D of human PrP (*red*), and D159N of dog PrP (*blue*) (*E*) were incubated in 1 × PBS (pH 7.4) containing 2 M GdnHCl with agitation at 220 rpm and with 2% (v/v) preformed seed fibrils of HoPrP-WT, H177R, CaPrP-WT, R177H, N159D, and D159N, respectively, at the initial time and then analyzed by ThT binding assay (*A*−*F*). *A*, *C*, and *E*, the ThT fluorescence intensity was expressed as the mean ± S.D. (with error bars) of values obtained in three independent experiments. The solid lines show the best exponential fit for the ThT intensity-time curves. *B*, *D*, and *F*, the fibril formation lag time in PrP, including HoPrP-WT, H177R, N159D, CaPrP-WT, R177H, and D159N, in the absence (*blue*) and presence of preformed seed fibrils (*red*) (*open black circles* shown in scatter plots) was determined by fitting ThT fluorescence intensity versus time to a sigmoidal equation and was expressed as the mean ± S.D. (with error bars) of values obtained in three independent experiments. HoPrP-WT + HoPrP-WT seeds, *p* = 0.00086; H177R + H177R seeds, *p* = 0.00026; CaPrP-WT + CaPrP-WT seeds, *p* = 0.00082; R177H + R177H seeds, *p* = 0.0000073; N159D + N159D seeds, *p* = 0.0016; and D159N + D159N seeds, *p* = 0.00093. *B* and *D*, H177R, *p* = 0.000034; H177R + H177R seeds, *p* = 0.000011; R177H, *p* = 0.0012; and R177H + R177H seeds, *p* = 0.00015. Statistical analyses were performed using the student *t*-test. Values of *p* < 0.05 indicate statistically significant differences. The following notation is used throughout: *, *p* < 0.05; **, *p* < 0.01; ***, *p* < 0.001; and ****, *p* < 0.0001 relative to control (the lag time for PrP in the absence of preformed seed fibrils, *B*, *D*, and *F*) or control (the lag time for wild-type PrP in the absence and presence of preformed seed fibrils, *B* and *D*).

**Figure S7. Arg177 from the dog PrP blocks the structural instability of human PrP, but His177 from the human PrP triggers the structural instability of dog PrP.** Samples (10 μM) of HoPrP-WT and its single variant H177R (*A* and *B*) as well as CaPrP-WT and its single variant R177H (*C* and *D*) were incubated in 1 × PBS (pH 7.4) at 25 °C (*red*) and 53 °C (*blue*) for 1 h, respectively, with agitation at 220 rpm and then analyzed by ThT binding assay (*A* and *C*) and ANS binding assay (*B* and *D*). The maximum ThT and ANS fluorescence intensities (*open black circles* shown in scatter plots) were determined and expressed as the mean ± S.D. (with error bars) of values obtained in three independent experiments. We have shown individual data points overlayed on the bar graphs. *A*, HoPrP-WT, *p* = 0.0023; H177R, *p* = 0.95. *B*, HoPrP-WT, *p* < 0.0001; H177R, *p* = 0.073. *C*, CaPrP-WT, *p* = 0.085; R177H, *p* < 0.0001. *D*, CaPrP-WT, *p* = 0.57; R177H, *p* < 0.0001. Statistical analyses were performed using the student *t*-test. Values of *p* < 0.05 indicate statistically significant differences. The following notation is used throughout: *, *p* < 0.05; **, *p* < 0.01; ***, *p* < 0.001; and ****, *p* < 0.0001 relative to control (the maximum ThT/ANS fluorescence intensity measured at 25 °C).

**Figure S8.** **Arg177 from the dog PrP strongly slows down the LLPS of GPI-anchored and glycosylated full-length human PrP^C^.** 10 (*A* and *E*), 20 (*B* and *F*), 30 (*C* and *G*), 40 (*D*, *H*, *I*, and *M*), 50 (*J* and *N*), 60 (*K* and *O*) or 70 (*L* and *P*) μM full-length wild-type human PrP^C^ (HoPrP-WT) (*A*−*H*) and its single variant H177R (*I*−*P*), both expressed and purified from sf9 insect cells, were labeled by TAMRA (*red fluorescence*) and incubated with 1 × PBS (pH 7.4) containing 12.5% (w/v) PEG 8000 on ice to induce LLPS for 10 min. Liquid droplets of the human PrP^C^ were observed by confocal microscopy, with excitation at 561 nm. *A*−*D* and *I*−*L*, TAMRA images; *E*−*H* and *M*−*P*, brightfield images. The scale bar represents 10 μm.

**Figure S9. Arg177 and Asp159 from the dog PrP significantly inhibit the aggregation of human PrP in cell cultures, but His177 and Asn159 from the human PrP greatly enhance the aggregation of dog PrP in cell cultures.** HEK-293T cells stably expressing full-length wild-type human PrP^C^ (HoPrP-WT), H177R PrP^C^ or N159D PrP^C^ (*A*) and MDCK cells stably expressing full-length wild-type dog PrP^C^ (CaPrP-WT), R177H PrP^C^ or D159N PrP^C^ (*B*) were cultured for 2 days. *A* and *B*, the sarkosyl-insoluble pellets from the above cells were probed using the anti-PrP monoclonal antibody 3F4, and the corresponding cell lysates were probed using 3F4 and anti-β-actin antibody, respectively. Marks at the left of the gels indicate the positions of the molecular weight markers. *C* and *D*, the normalized amount of insoluble PrP aggregates in HEK-293T cells overexpressing human PrP^C^ (*C*) or MDCK cells overexpressing dog PrP^C^ (*D*) (*open black circles* shown in scatter plots) was determined as a ratio of the density of insoluble PrP aggregate bands over that of the total PrP bands in cell lysates and expressed as mean ± S.D. (with error bars) of values obtained in three independent experiments. *C*, H177R, *p* = 0.00018; N159D, *p* = 0.014. *D*, R177H, *p* = 0.00085; D159N, *p* = 0.012. HEK-293T cells overexpressing HoPrP-WT (*C*) or MDCK cells overexpressing CaPrP-WT (*D*) were used as a control. Statistical analyses were performed using the student *t*-test. Values of *p* < 0.05 indicate statistically significant differences. The following notation is used throughout: *, *p* < 0.05; **, *p* < 0.01; and ***, *p* < 0.001 relative to control.

**Figure S1**


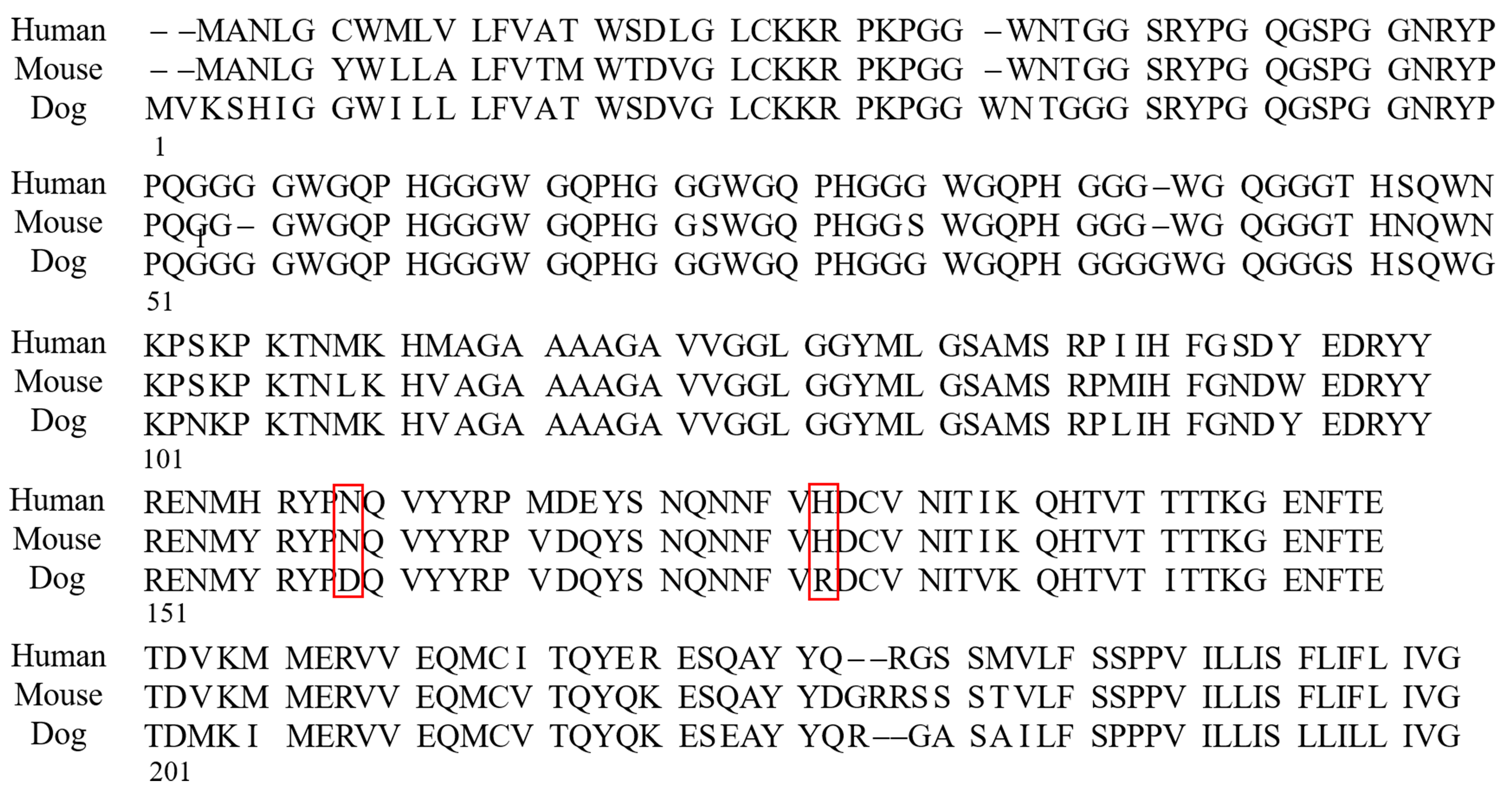


**Figure S2**


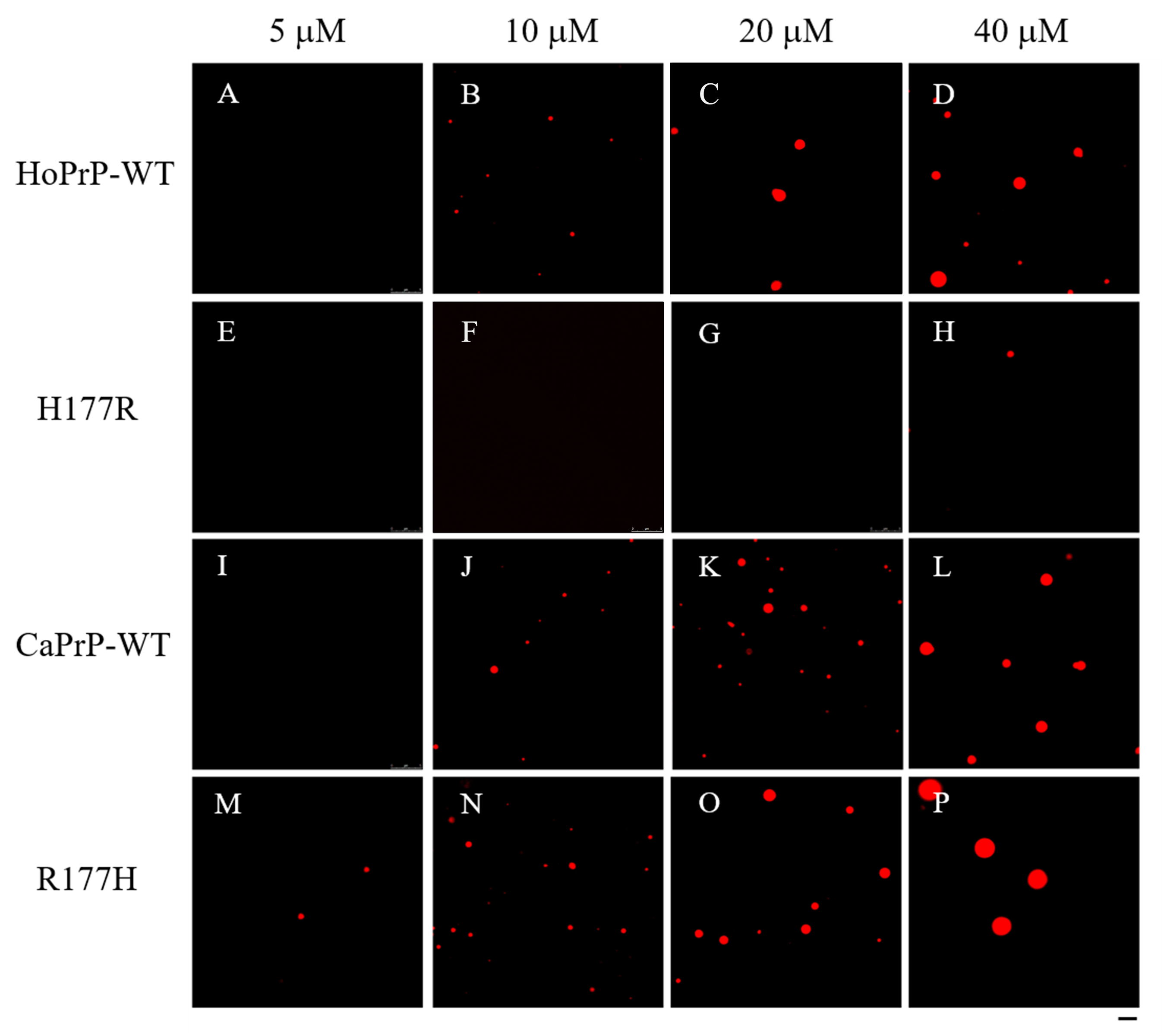


**Figure S3**


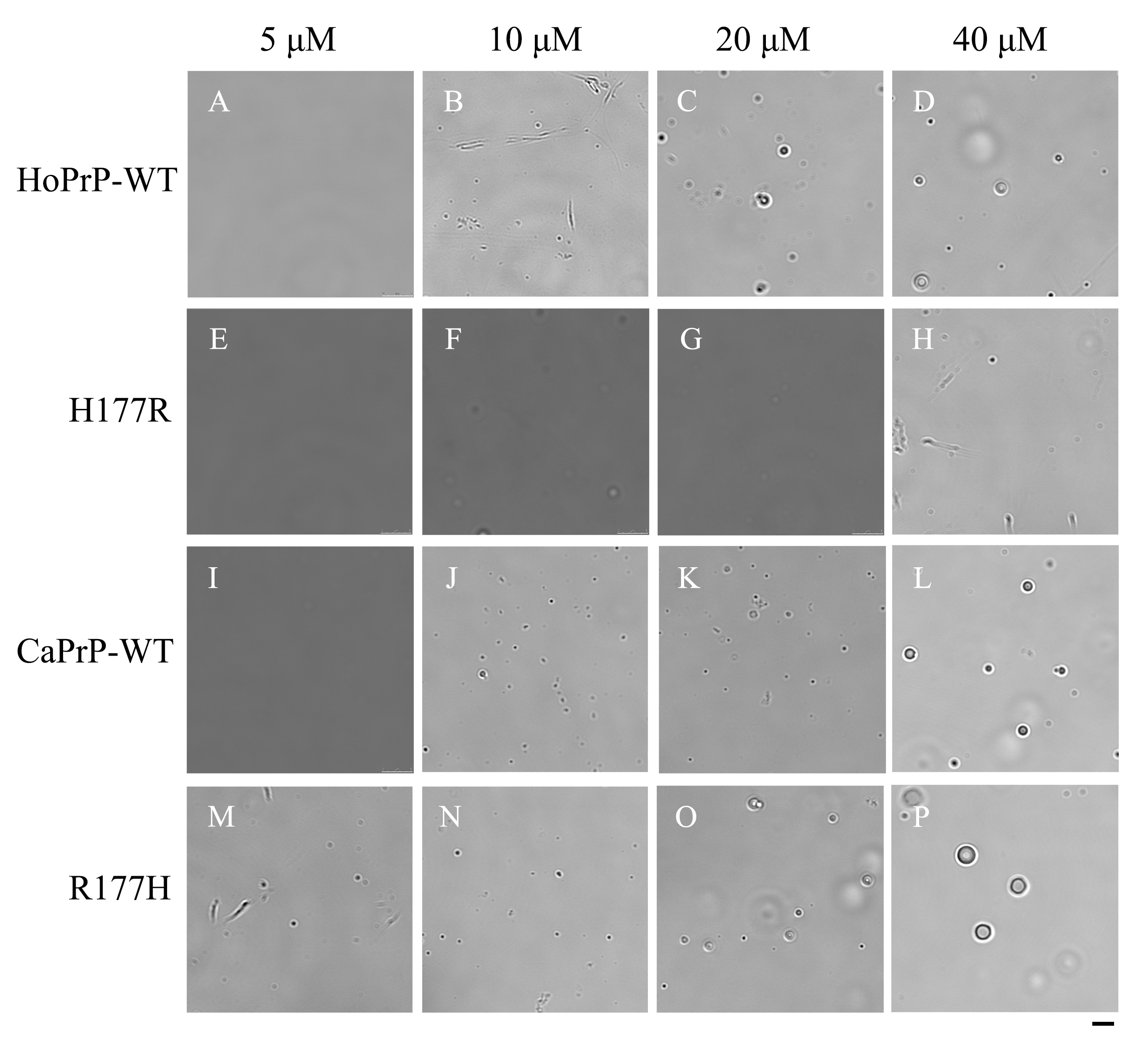


**Figure S4**


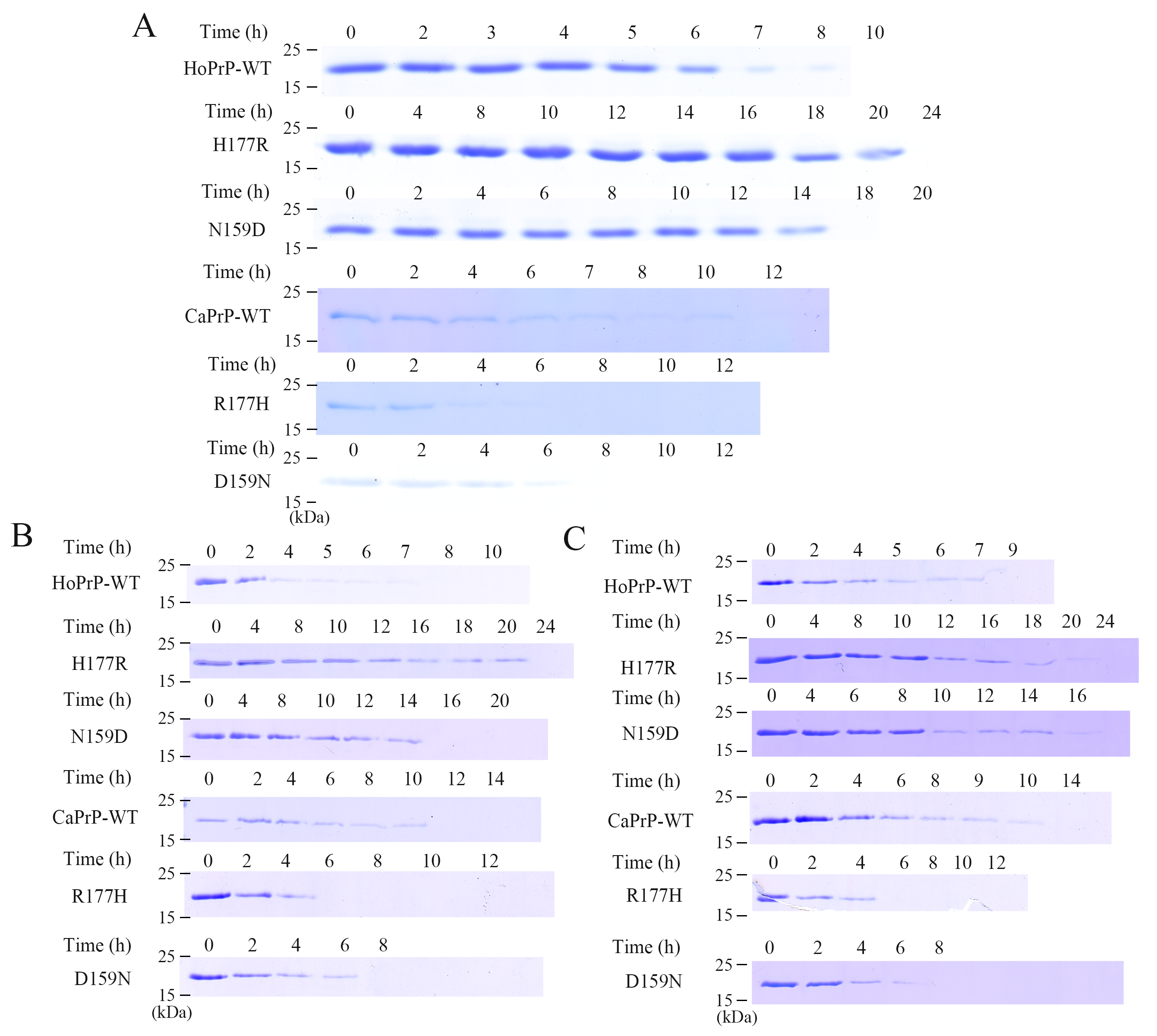


**Figure S5**


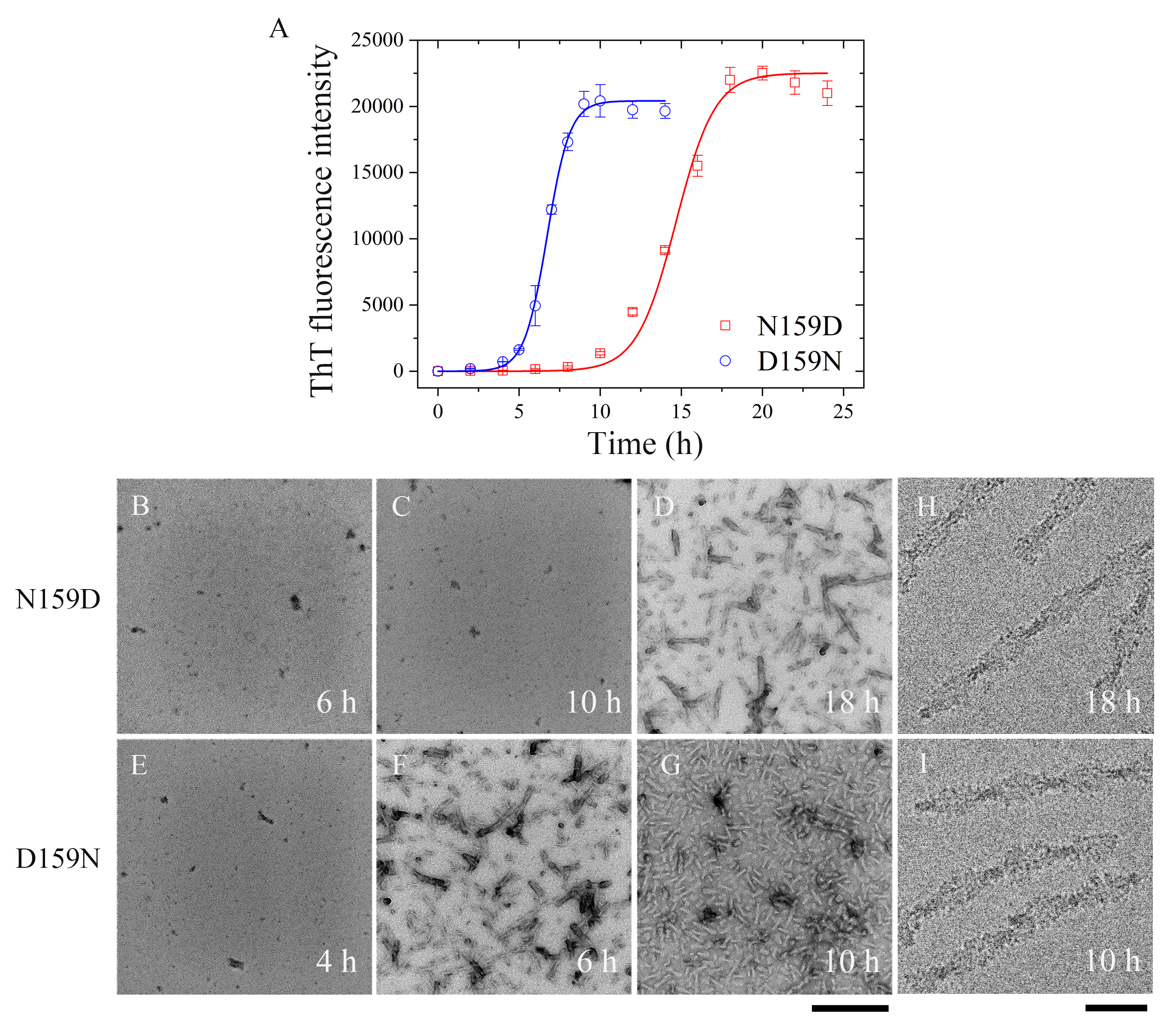


**Figure S6**


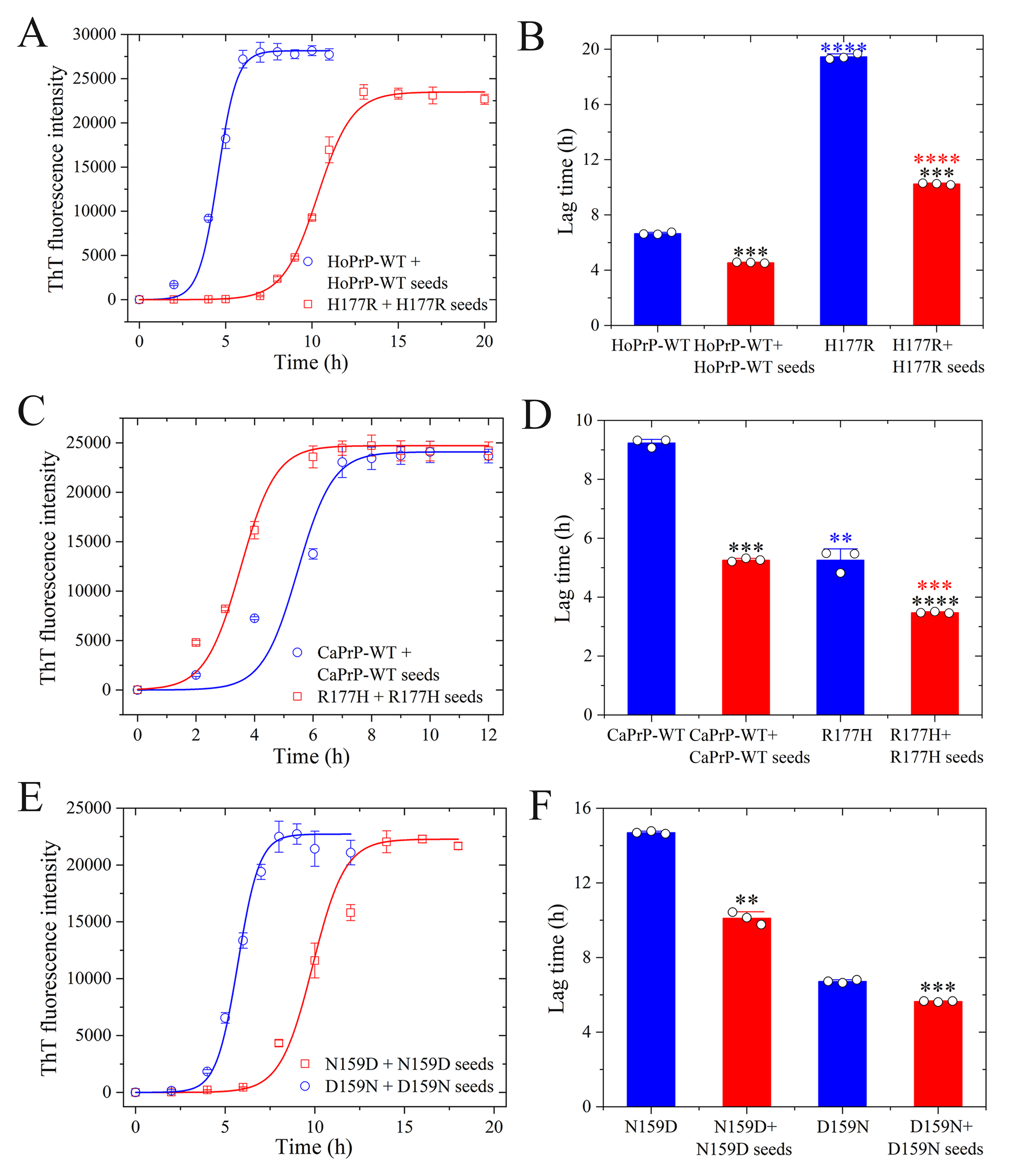


**Figure S7**


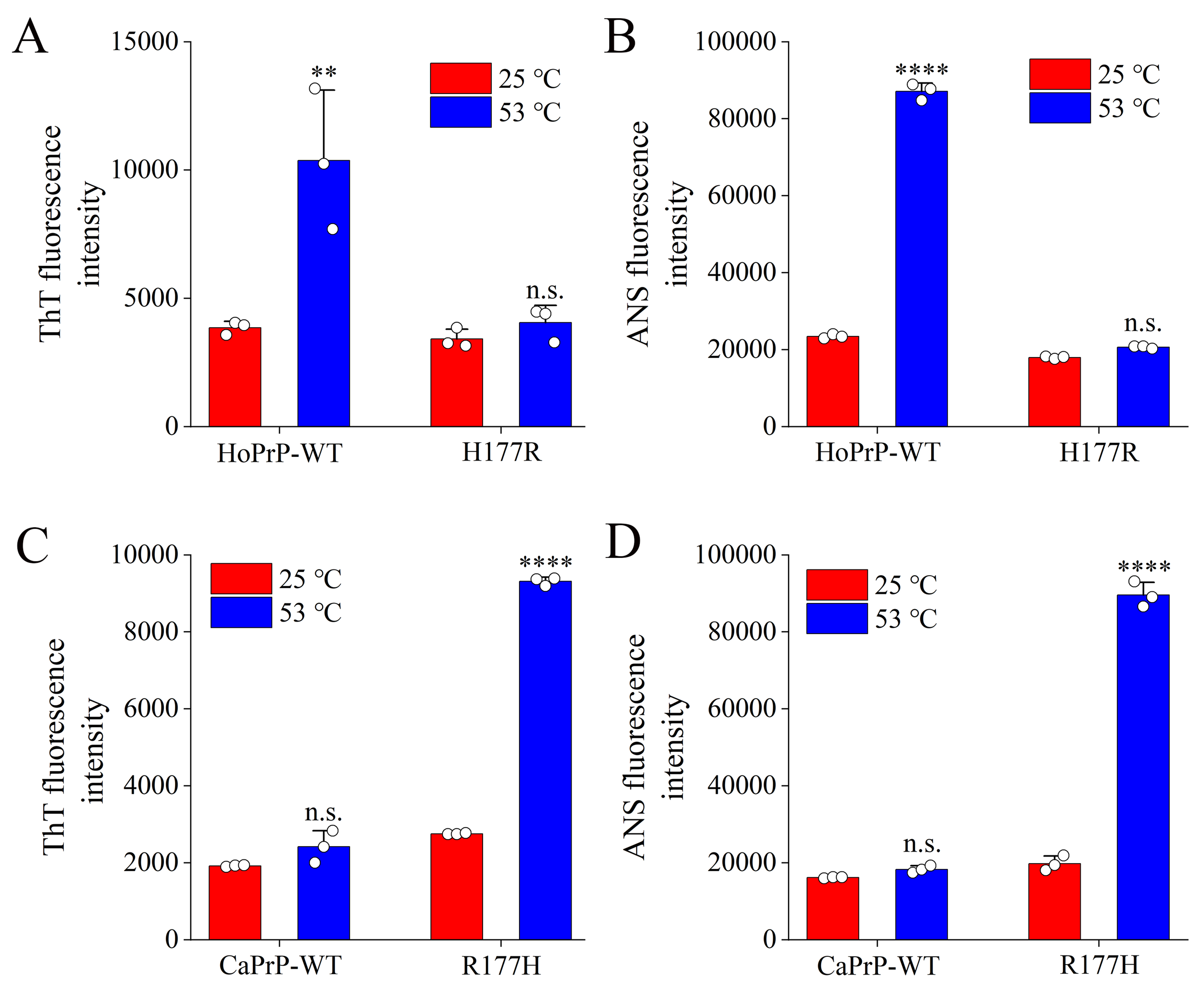


**Figure S8**


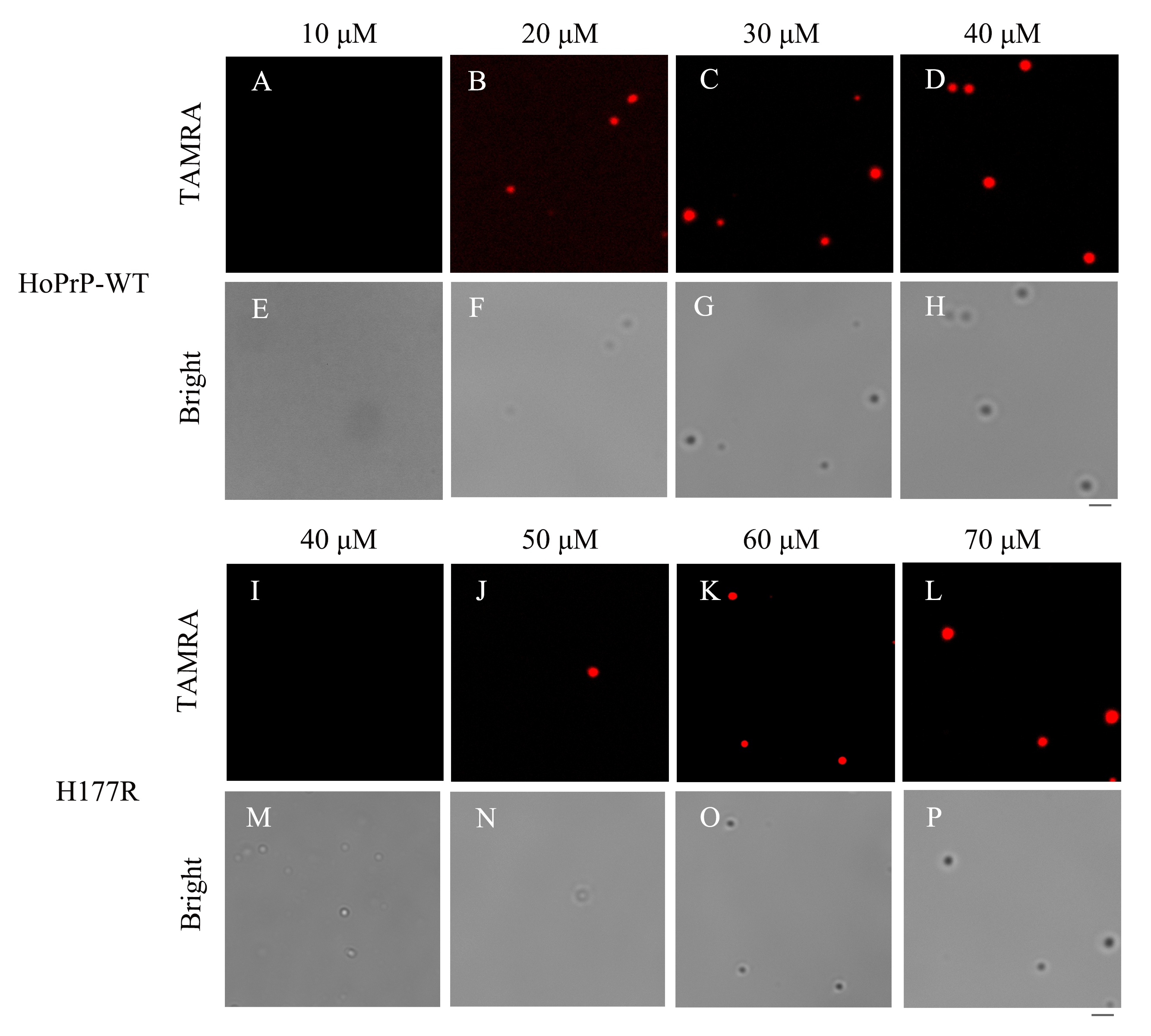


**Figure S9**


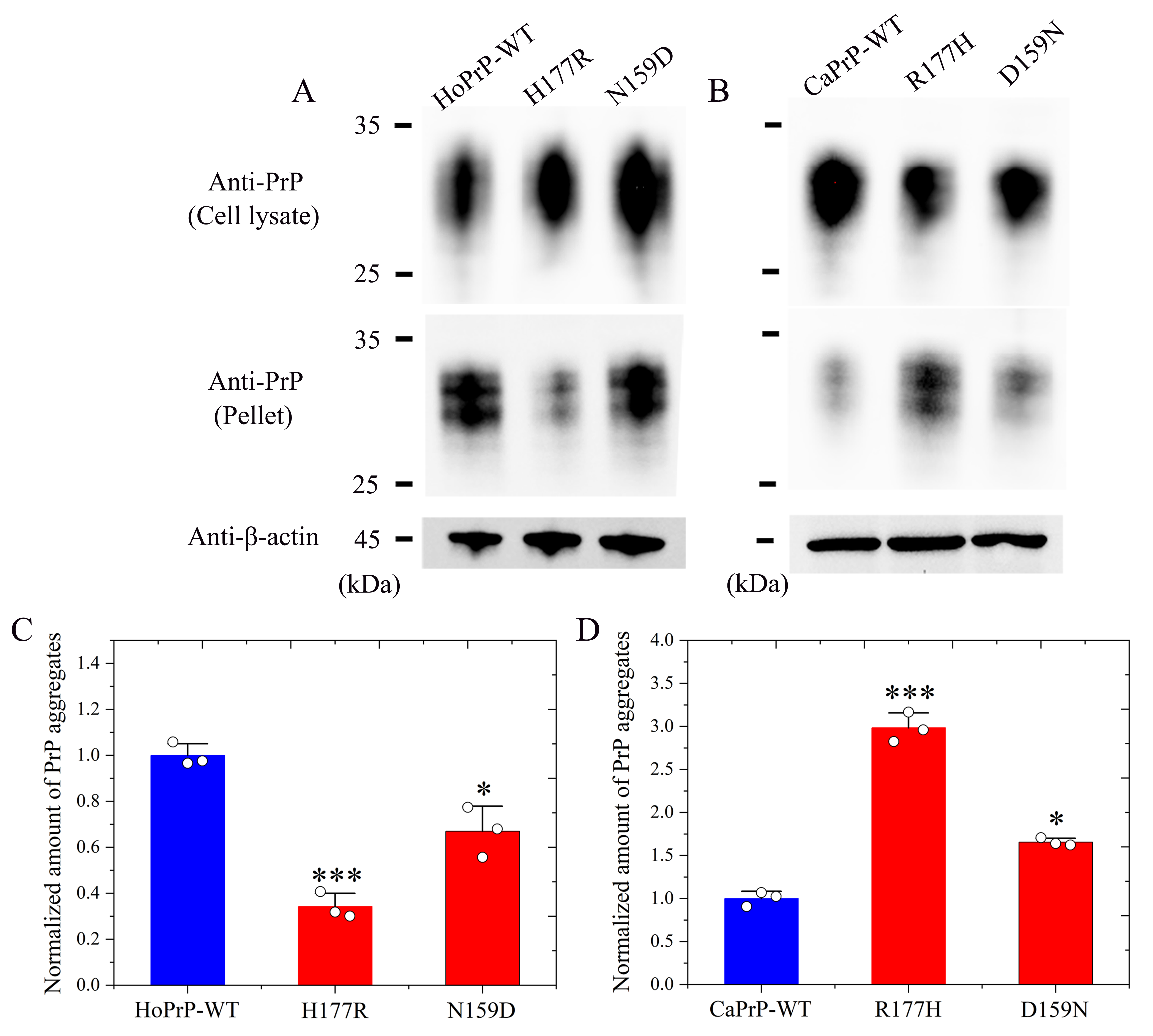

Supplement: Supporting Table S1 and Figures S1–S9 [file mmc1.doc]
